# Diagnostic and Prognostic Value of Blood and Cerebrospinal Fluid Biomarkers in Amyotrophic Lateral Sclerosis: A Systematic Review and Meta‐Analysis

**DOI:** 10.1111/ene.70382

**Published:** 2025-10-27

**Authors:** Kazuki Obara, Daisuke Ito, Christer Nilsson, Shorena Janelidze, Alexander Santillo, Masahisa Katsuno, Niklas Mattsson‐Carlgren

**Affiliations:** ^1^ Clinical Memory Research Unit, Department of Clinical Sciences Malmö Lund University Lund Sweden; ^2^ Department of Neurology Nagoya University Graduate School of Medicine Nagoya Aichi Japan; ^3^ Department of Clinical Research Education Nagoya University Graduate School of Medicine Nagoya Aichi Japan; ^4^ Department of Neurology and Rehabilitation Medicine Skåne University Hospital Lund Sweden; ^5^ Memory Clinic Skåne University Hospital Malmö Sweden; ^6^ Wallenberg Center for Molecular Medicine Lund University Lund Sweden

**Keywords:** amyotrophic lateral sclerosis, biomarker, diagnosis, disease progression, neurofilament proteins, prognosis, survival

## Abstract

**Background:**

Reliable biomarkers for amyotrophic lateral sclerosis (ALS) are urgently needed due to diagnostic and prognostic challenges. This systematic review and meta‐analysis aimed to synthesize recent evidence on the utility of blood and cerebrospinal fluid (CSF) biomarkers for ALS.

**Methods:**

We systematically reviewed studies published from January 1, 2019 to March 25, 2025, that evaluated blood or CSF biomarkers for ALS. Eligible studies reported diagnostic performance, group‐level biomarker values, hazard ratios (HRs) for survival, or correlations with functional rating scales or disease progression rates. Study quality was assessed using the QUADAS‐2 and QUIPS frameworks. Random‐effects models were employed to pool summary receiver operating characteristic (SROC) curves, HRs, standardized mean differences, and correlation coefficients.

**Results:**

We included 47 studies in the SROC analysis and 27 in the HR analysis, covering 9078 participants (5556 ALS and 3522 controls). Neurofilament light chain (NfL) consistently demonstrated the highest diagnostic accuracy (sensitivity/specificity: 0.81–0.87 vs. ALS mimics) and high prognostic value (pooled HRs: 2.8–4.3) in both blood and CSF. CSF chitinases and the p‐tau/t‐tau ratio showed moderate utility. Other biomarkers, including interleukins, had limited clinical relevance. Most studies showed moderate to high risk of bias, with methodological heterogeneity and limited transparency.

**Conclusions:**

NfL is the most validated biomarker for ALS diagnosis and prognosis, in both blood and CSF. However, its limited accuracy when used alone carries a considerable risk of misclassification. Future studies should adopt prevalence‐specific strategies and integrate biomarkers within multimodal frameworks to enhance diagnostic and prognostic precision.

**Abbreviations**
AD: Alzheimer's disease
ALS: amyotrophic lateral sclerosis
AUC: area under the curve
Aβ42: amyloid‐beta 1–42
CHI3L2: chitinase‐3‐like protein 2
CHIT1: chitotriosidase 1
CK: creatine kinase
CSF: cerebrospinal fluid
CysC: cystatin C
DC: neurological disease controls
FTD: frontotemporal dementia
GFAP: glial fibrillary acidic protein
IL‐10: interleukin‐10
IL‐2: interleukin‐2
IL‐6: interleukin‐6
MCP1: monocyte chemoattractant protein‐1
NfH: neurofilament heavy chain
NfL: neurofilament light chain
NHC: neurologically healthy controls
NLR: neutrophil‐to‐lymphocyte ratio
NPV: negative predictive value
pNfH: phosphorylated neurofilament heavy chain
PPV: positive predictive value
PRISMA: preferred reporting items for systematic reviews and meta‐analyses
p‐tau181: phosphorylated tau at threonine 181
QUADAS‐2: Quality Assessment of Diagnostic Accuracy Studies 2
QUIPS: Quality in Prognosis Studies
ROC: receiver operating characteristic
sEV‐TDP43: small extracellular vesicle TAR DNA‐binding protein 43
SMD: standardized mean difference
SPP1: secreted phosphoprotein 1
sTREM2: soluble triggering receptor expressed on myeloid cells 2
TDP‐43: TAR DNA‐binding protein 43
TNF‐α: tumor necrosis factor‐alpha
t‐tau: total tau
UA: uric acid
UCHL1: ubiquitin carboxyl‐terminal hydrolase L1
YKL40: chitinase‐3‐like protein 1

## 1 Introduction

Amyotrophic lateral sclerosis (ALS) is a neurodegenerative disorder characterized by the progressive loss of upper and lower motor neurons. Approximately 90% of ALS cases are sporadic, and the underlying mechanisms of disease development remain unclear [1]. As global populations age, the incidence and prevalence of ALS are increasing [2]. The available therapies are not curative [3, 4, 5], and the design of clinical trials is complicated by the heterogeneity of disease presentation and progression, which makes both diagnosis and prognosis challenging [6]. Historically, the diagnosis of sporadic ALS has been primarily based on clinical assessment and the exclusion of alternative conditions. This process is often time‐consuming, costly, and results in varying degrees of diagnostic certainty (e.g., possible, probable, and definite) [7]. More recently, the Gold Coast criteria removed diagnostic categories to improve sensitivity and facilitate earlier diagnosis [8, 9]. However, diagnostic precision continues to rely heavily on the expertise of the evaluating neurologist and neurophysiologist, and the shift toward prioritizing sensitivity can increase the risk of false positives, particularly at early stages of disease [10, 11]. This persistent risk of misclassification complicates the early identification of ALS, which is crucial for developing new treatments. Drug development in other neurodegenerative diseases has benefited from objective biomarkers that can diagnose and prognosticate with better accuracy than clinical evaluation (e.g., Alzheimer's disease) [12, 13]. There should be potential for a similar development in ALS, driven by the availability of high‐performing fluid biomarkers.

Neurofilaments have gained attention as promising diagnostic and prognostic biomarkers and are increasingly used as supportive measures in ALS clinical trials [5, 14, 15]. However, since the release of neurofilament is a nonspecific process, the levels can be elevated in several neurodegenerative diseases [16], limiting their utility in distinguishing ALS from mimicking conditions [17]. Other fluid biomarkers, such as chitinases, p‐tau/t‐tau ratio, and small extracellular vesicle TAR DNA‐binding protein 43 (sEV‐TDP43), have shown promise in other studies [18, 19, 20, 21, 22]. Yet, their diagnostic and prognostic performance across studies remains to be systematically assessed. Previous systematic reviews and meta‐analyses have primarily focused on each biomarker category [23, 24, 25, 26, 27, 28]. Systematic comparison of diverse ALS biomarkers is essential to determine their diagnostic and prognostic relevance. Due to the development of both analytical techniques and ALS clinical diagnostic systems, such syntheses should focus on recent literature that reflects current methodologies and clinical practice.

The aim of this study is to synthesize recent evidence on the utility of blood and cerebrospinal fluid (CSF) biomarkers for ALS. We conducted a systematic review and meta‐analysis to evaluate the diagnostic and prognostic value of blood and CSF biomarkers, as well as their correlation with functional rating scales.

## 2 Methods

### 2.1 Study Selection

#### 2.1.1 Data Source and Search Strategy

We conducted a systematic literature search across the following databases to identify relevant studies published up to March 25, 2025: PubMed/MEDLINE, Embase, Web of Science, CINAHL, Cochrane Library, and PsycINFO. The search strategy incorporated terms related to ALS, biomarkers, blood, CSF, and diagnostic or prognostic assessments. The list of all database search strategies is described in detail in Appendix S1. This study's protocol was registered with PROSPERO (CRD42024577152) and was conducted in accordance with the Preferred Reporting Items for Systematic Reviews and Meta‐Analyses (PRISMA).

#### 2.1.2 Eligibility Criteria

Studies published after January 1, 2019, were included if they met the following criteria: (1) evaluated blood or CSF biomarkers in ALS patients and (2) reported at least one of the following: (a) summary statistics of biomarker levels for both ALS and control groups (e.g., mean and SD), (b) diagnostic performance, including both sensitivity and specificity, (c) hazard ratios (HRs) from univariable or multivariable Cox proportional hazards models, (d) Spearman's correlation coefficients between biomarker levels and baseline ALS Functional Rating Scale (ALSFRS; original or revised version), or (e) Spearman's correlation coefficients between biomarker levels and disease progression rate (DPR).

Studies were excluded if they were case reports, small sample size studies (including fewer than 10 ALS cases), conference presentations, non‐systematic reviews, or non‐English publications. The eligibility of each study was assessed independently by KO and NMC using the Covidence online tool. In cases of disagreement, consensus was reached through discussion.

### 2.2 Outcome Assessment

In this study, we evaluated each biomarker based on two primary outcomes: diagnostic value and prognostic value.

#### 2.2.1 Diagnostic Value Assessment

For the diagnostic value assessment, we primarily pooled sensitivity, specificity, and the area under the receiver operating characteristic (ROC) curve (AUC). Comparison groups were categorized as follows: (1) neurologically healthy controls (NHC): primarily comprising healthy individuals, though some studies also included individuals with non‐neurological conditions; (2) ALS mimics: patients with conditions presenting with muscle weakness or initially suspected ALS who were later excluded during follow‐up; and (3) neurological disease controls (DC): patients with neurological diseases not primarily involving muscle weakness, such as Alzheimer's disease (AD) and Parkinson's disease. As a secondary analysis, standardized mean differences (SMDs) were calculated for each biomarker.

#### 2.2.2 Prognostic Value Assessment

To assess the prognostic value, we primarily pooled HRs from Cox proportional hazards models predicting time to death or tracheostomy. We extracted HRs separately for univariable and multivariable models and documented the covariates included in the multivariable analyses.

As a supplementary analysis, we evaluated the correlation of biomarkers with functional measures. Specifically, we examined (1) the correlation with the functional rating scale (ALSFRS or ALSFRS‐R) at baseline and (2) the correlation with the DPR at baseline, calculated as (48—ALSFRS‐R) / months from onset or (40—ALSFRS)/months from onset.

### 2.3 Quality Assessment

For diagnostic studies included in the meta‐analysis, the risk of bias (RoB) was evaluated using the Quality Assessment of Diagnostic Accuracy Studies‐2 (QUADAS‐2) [29] protocol by KO and DI independently. In cases of disagreement, consensus was reached through discussion. For prognostic studies, the same procedure was applied using the Quality in Prognosis Studies (QUIPS) [30] tool. The detailed protocol is provided in the Appendices S2 and S3.

### 2.4 Statistical Analysis

#### 2.4.1 Meta‐Analysis of Diagnostic Values

Studies reporting the sensitivity and specificity of ALS diagnosis were included in this analysis. A bivariate random‐effects model, as proposed by Reitsma et al. [31], was applied to obtain pooled estimates of sensitivity, specificity, and AUC. Corresponding hierarchical summary ROC curves were generated for the analysis. A univariate approach with a random‐effects model was also applied to pool the reported AUC values as part of the sensitivity analysis.

As a secondary analysis, standardized mean differences (SMDs) were calculated for biomarker concentrations reported as means and SDs or medians and interquartile ranges (IQRs). If only medians and IQRs were available, values were approximated accordingly by the R package *meta*. To avoid duplication, when multiple studies involved the same cohort for a given biomarker, only the one with the largest sample size was included.

#### 2.4.2 Meta‐Analysis of Prognostic Values

For the prognostic meta‐analyses, random‐effects models were applied to pool HRs for time to death or tracheostomy. Studies were included in these analyses if they reported HRs based on dichotomized biomarker levels (e.g., above vs. below median, high vs. low tertile groups). Studies reporting HRs based on continuous biomarker levels were excluded, as meta‐analysis was not feasible due to substantial heterogeneity in scaling methods (e.g., per raw unit, per SD, or log transformations [log_2_, log_e_, log_10_]). HRs from univariable and multivariable models were synthesized separately, with covariates used in each study collected.

As a supplementary analysis, we synthesized studies reporting Spearman's correlation coefficients between biomarker levels and either ALSFRS (original or revised) or DPR, using random‐effects models.

#### 2.4.3 Heterogeneity and Reporting Bias Assessment

For each meta‐analysis, heterogeneity was assessed using Cochran's *Q* test, the *I* ^2^ index, and the *τ* ^2^ value. Reporting bias was assessed using funnel plots and Egger's test for biomarkers with 10 or more studies. For those with fewer than 10 studies, such assessments were not feasible, and the possibility of reporting bias cannot be excluded [32]. All statistical analyses were conducted using R version 4.5.0. Functions from the packages *meta* (‘metacont’, ‘metacorr’, ‘metagen’, ‘metabias’) and *mada* (‘reitsma’) were employed.

## 3 Results

### 3.1 Overview

A PRISMA flowchart illustrating the study selection process is provided in Figure S1. A total of 3065 studies were identified through the systematic search, and 479 remained after duplicate removal and title/abstract screening. Of these, 321 met the inclusion criteria for data extraction. Only biomarkers with available data from at least two independent study cohorts were included in the meta‐analyses. Table 1 provides an overview of the evaluated biomarkers. All eligible studies and their characteristics are detailed in Appendix S4 (Excel file).

**TABLE 1:** Overview of biomarkers included in the systematic review.

Associated pathology		Diagnostic value (see Tables 2 and S1)				Prognostic value (see Tables 3 and 4)			
		Blood		CSF		Blood		CSF	
		AUC	|SMD|	AUC	|SMD|	HR	Corr. with DPR	HR	Corr. with DPR
**Neurodegeneration**									
NfL	Axonal injury	High	High	High	High	High	Moderate	High	Moderate
pNfH	Axonal injury	Moderate	Moderate to high	High	High	High	Moderate	High	Moderate
NfH	Axonal injury^e^	Moderate	High	Moderate	High	NA^b^	Weak	NA^b^	Moderate
p‐tau181	Tau phosphorylation; neurodegeneration	NA^a^	Moderate to high	Moderate	Uncertain	NA^b^	NA^d^	NA^b^	NA^d^
t‐tau	Neuronal damage and degeneration	NA^a^	Uncertain	Low	Uncertain	NA^b^	NA^d^	NA^b^	Weak
p‐tau/t‐tau	Relative degree of tau phosphorylation	NA^a^	NA^c^	Moderate	High	NA^b^	NA^d^	NA^b^	Weak
UCHL1	Neuronal injury	NA^a^	Uncertain	NA^a^	NA^c^	NA^b^	NA^d^	NA^b^	NA^d^
**Neuroinflammation**									
CHIT1	Glial and macrophage activation	NA^a^	Uncertain	Moderate	Moderate to high	NA^b^	NA^d^	Uncertain	Moderate
YKL40	Glial‐mediated inflammatory responses	NA^a^	Uncertain	Moderate	Moderate to high	NA^b^	NA^d^	Uncertain	Moderate
CHI3L2	Glial‐mediated inflammatory responses	NA^a^	NA^c^	NA^a^	Moderate to high	NA^b^	NA^d^	NA^b^	NA^d^
GFAP	Astrocyte activation	Low	Uncertain	NA^a^	Low	Uncertain	NA^d^	NA^b^	NA^d^
sTREM2	Microglial activation	NA^a^	Low to moderate	NA^a^	Low to moderate	NA^b^	NA^d^	NA^b^	NA^d^
**Proteinopathy**									
TDP‐43	Hallmark of ALS pathology	NA^a^	NA^c^	Uncertain	NA^c^	NA^b^	NA^d^	NA^b^	NA^d^
sEV‐TDP‐43	TDP‐43 detected in small extracellular vesicles	NA^a^	High	NA^a^	NA^c^	NA^b^	NA^d^	NA^b^	NA^d^
Aβ42	Amyloid‐beta co‐pathology	NA^a^	NA^c^	NA^a^	Uncertain	NA^b^	NA^d^	NA^b^	NA^d^
**Systemic inflammation**									
CRP	Systemic inflammation	NA^a^	NA^c^	NA^a^	NA^c^	Low	NA^d^	NA^b^	NA^d^
Interleukins	Immune signaling	NA^a^	Uncertain (IL‐6)	NA^a^	NA^c^	NA^b^	Weak or uncertain (IL‐2,6,10)	NA^b^	NA^d^
TNF‐α	Inflammation and neurotoxicity	NA^a^	NA^c^	NA^a^	NA^c^	NA^b^	Weak	NA^b^	NA^d^
SPP1	Immune modulation	NA^a^	Uncertain	NA^a^	NA^c^	NA^b^	NA^d^	NA^b^	NA^d^
MCP1	Monocyte recruitment	NA^a^	NA^c^	Uncertain	NA^c^	NA^b^	NA^d^	Uncertain	Weak
NLR	Systemic inflammation	NA^a^	Low	NA^a^	NA^c^	Uncertain	NA^d^	NA^b^	NA^d^
**Muscle**									
Creatinine	Muscle volume and kidney function	NA^a^	Moderate to high	NA^a^	NA^c^	Uncertain	Weak	NA^b^	NA^d^
CK	Muscle injury	NA^a^	NA^c^	NA^a^	NA^c^	NA^b^	Weak	NA^b^	NA^d^
**Metabolism**									
Ghrelin	Energy metabolism	NA^a^	Low	NA^a^	NA^c^	NA^b^	NA^d^	NA^b^	NA^d^
Leptin	Energy metabolism	NA^a^	Uncertain	NA^a^	NA^c^	NA^b^	NA^d^	NA^b^	NA^d^
UA	Oxidative stress	NA^a^	NA^c^	NA^a^	NA^c^	Uncertain	NA^d^	NA^b^	NA^d^
**Kidney function**									
Cystatin C	Kidney filtration function	NA^a^	NA^c^	NA^a^	NA^c^	Low	NA^d^	NA^b^	NA^d^

*Note: Interpretation thresholds*: AUC: high ≥ 0.90; moderate 0.70–0.89; low < 0.70; uncertain if 95% CI includes 0.5. |SMD|: high ≥ 1.0; moderate to high 0.75–0.99; low to moderate 0.50–0.74; low < 0.50; uncertain if 95% CI includes 0. HR: high if HR ≥ 2.0 or ≤ 0.5 with 95% CI excluding 1; low if HR 1.0–2.0 or 0.5–1.0 with 95% CI excluding 1; uncertain if 95% CI includes 1. Corr.: moderate |ρ| 0.25–0.5; weak |ρ| < 0.25; uncertain if 95% CI includes 0.

Abbreviations: AUC, area under the receiver operating characteristic curve; Aβ42, amyloid beta 1–42; CHI3L2, chitinase‐3‐like protein 2; CHIT1, chitinase‐1; CI, confidence interval; CK, creatine kinase; corr., correlation; CRP, C‐reactive protein; CSF, cerebrospinal fluid; DPR, disease progression rate; GFAP, glial fibrillary acidic protein; HR, hazard ratio; IL, interleukin; MCP1, monocyte chemoattractant protein‐1; NA, not analyzed; NfH, neurofilament heavy chain; NfL, neurofilament light chain; NLR, neutrophil‐to‐lymphocyte ratio; pNfH, phosphorylated neurofilament heavy chain; p‐tau 181, phosphorylated tau 181; sEV‐TDP‐43, small extracellular vesicle TDP‐43; SMD, standardized mean difference; SPP1, secreted phosphoprotein 1 (osteopontin); sTREM2, soluble triggering receptor expressed on myeloid cells 2; TDP‐43, TAR DNA‐binding protein 43; TNF‐α, tumor necrosis factor alpha; t‐tau, total tau; UA, uric acid; UCHL1, ubiquitin carboxyl‐terminal hydrolase L1; YKL40, chitinase‐3‐like protein 1.

^a^ Summary ROC meta‐analysis not feasible (insufficient eligible studies or incompatible data).

^b^ HR meta‐analysis not feasible (insufficient eligible studies or incompatible data).

^c^ SMD meta‐analysis not feasible (insufficient eligible studies or incompatible data).

^d^ Correlation with DPR meta‐analysis not feasible (insufficient eligible studies or incompatible data).

^e^ Alternative assay to pNfH.

### 3.2 Diagnostic Values

A total of 47 studies met the inclusion criteria for the SROC meta‐analysis. These studies included 5556 ALS patients and 3522 controls (including 817 ALS mimics, 1776 NHC, and 929 DC). NfL was assessed in the majority of these studies (34/47, 72%), reflecting its prominence in the field.

#### 3.2.1 Summary ROC Analyses

**FIGURE 1:**
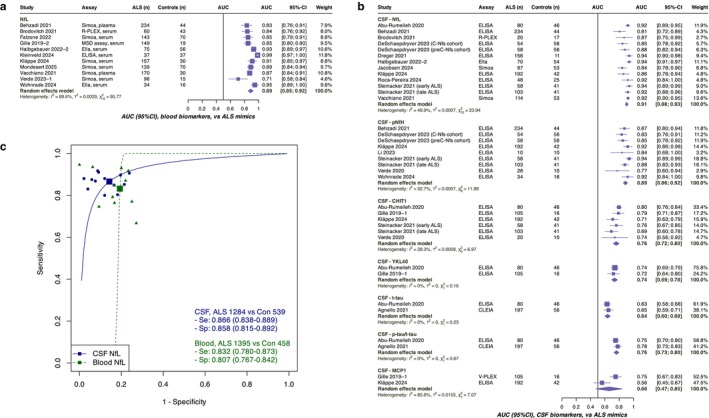
Diagnostic Performance for Differentiating ALS from ALS Mimics (Blood and CSF Biomarkers). Panel (a) shows the pooled AUCs for blood biomarkers, and panel (b) shows those for CSF biomarkers, both estimated using random‐effects models. Panel (c) presents hierarchical summary ROC curves based on the Reitsma model. The blue curve represents CSF NfL, and the green curve represents blood NfL. Each square indicates the pooled sensitivity and specificity. Each blue circle represents an individual study reporting the diagnostic performance of CSF NfL, and each green triangle represents a study reporting that of blood NfL. The full list of included studies is provided in Appendix S4. ALS, amyotrophic lateral sclerosis; AUC, area under the curve; CHIT1, chitotriosidase 1; CSF, cerebrospinal fluid; MCP1, monocyte chemoattractant protein‐1; NfL, neurofilament light chain; pNfH, phosphorylated neurofilament heavy chain; ROC, receiver operating characteristic; YKL40, chitinase‐3‐like protein 1.

Pooled diagnostic performance for distinguishing ALS from NHC and ALS mimics is provided in Table 2. Overall, diagnostic performance was higher when distinguishing ALS from NHC than from ALS mimics, and CSF biomarkers generally outperformed blood biomarkers. The corresponding forest plots and SROC curves for the AUC are presented in Figure 1 and Figures S2 and S3.

**TABLE 2:** Summary of pooled diagnostic performance.

Biomarker	vs. *Neurologically Healthy Controls*							vs. *ALS mimics*						
	Cohorts	N, ALS	N, Con	Summary Se.*	Summary Sp.*	sAUC^†^	AUC (RE model)^‡^	Cohorts	N, ALS	N, Con	Summary Se.*	Summary Sp.*	sAUC^†^	AUC (RE model)^‡^
**Blood**														
NfL	11^¶^	1659	1214	0.90 (0.85, 0.94)	0.91 (0.86, 0.95)	0.96	0.96 (0.93, 0.98)	12^¶^	1395	458	0.83 (0.78, 0.87)	0.81 (0.77, 0.84)	0.81	0.89 (0.85, 0.92)
pNfH	3	1013	790	0.71 (0.67, 0.76)	0.86 (0.75, 0.93)	0.78	0.85 (0.81, 0.88)	—	—	—	—	—	—	—
NfH	2^§^	122	144	0.63 (0.28, 0.88)	0.85 (0.75, 0.92)	0.86	0.80 (0.61, 1.00)	—	—	—	—	—	—	—
GFAP	2	257	83	0.46 (0.19, 0.75)	0.75 (0.47, 0.91)	0.66	0.59 (0.52, 0.66)	—	—	—	—	—	—	—
**CSF**														
NfL	10	787	340	0.90 (0.85, 0.93)	0.88 (0.83, 0.93)	0.95	0.93 (0.89, 0.97)	13	1284	539	0.87 (0.84, 0.89)	0.86 (0.81, 0.89)	0.92	0.91 (0.88, 0.93)
pNfH	4	385	127	0.89 (0.83, 0.93)	0.96 (0.83, 0.99)	0.94	0.93 (0.88, 0.98)	9	771	316	0.86 (0.81, 0.91)	0.85 (0.81, 0.89)	0.87	0.88 (0.85, 0.91)
NfH	2	87	82	0.75 (0.31, 0.95)	0.86 (0.69, 0.94)	0.88	0.87 (0.67, 1.00)	—	—	—	—	—	—	—
CHIT1	6^§^	537	251	0.80 (0.72, 0.86)	0.84 (0.78, 0.89)	0.89	0.88 (0.81, 0.95)	6	558	196	0.72 (0.58, 0.83)	0.73 (0.55, 0.86)	0.78	0.76 (0.72, 0.81)
YKL40	2^§^	198	84	0.74 (0.56, 0.87)	0.75 (0.23, 0.97)	0.79	0.72 (0.40, 1.00)	2	185	62	0.71 (0.64, 0.77)	0.64 (0.52, 0.75)	0.72	0.74 (0.69, 0.78)
t‐tau	2	134	101	0.82 (0.64, 0.92)	0.63 (0.53, 0.72)	0.64	0.74 (0.70, 0.78)	2	277	102	0.68 (0.47, 0.84)	0.55 (0.45, 0.65)	0.57	0.64 (0.60, 0.68)
p‐tau181	3^§^	287	117	0.62 (0.32, 0.84)	0.76 (0.35, 0.95)	0.73	0.62 (0.53, 0.70)	—	—	—	—	—	—	—
p‐tau/t‐tau	2^§^	277	78	0.80 (0.72, 0.86)	0.78 (0.57, 0.91)	0.84	0.83 (0.79, 0.88)	2	277	102	0.76 (0.65, 0.84)	0.74 (0.64, 0.83)	0.80	0.76 (0.73, 0.80)
TDP‐43	2	98	88	0.88 (0.56, 0.97)	0.54 (0.14, 0.89)	0.82	0.67 (0.45, 0.89)	—	—	—	—	—	—	—
MCP1	—	—	—	—	—	—	—	2	297	58	0.89 (0.76, 0.96)	0.38 (0.18, 0.63)	0.74	0.66 (0.47, 0.85)

* Summary points on the hierarchical summary ROC curve, estimated from a bivariate random‐effects model (Reitsma model), with 95% confidence intervals (CIs).

^†^ sAUC: Area under the hierarchical summary ROC curve, estimated from the Reitsma model. 95% CIs for sAUC are not provided by the ‘mada’ package.

^‡^ AUC (RE model): Pooled AUC derived from a univariate random‐effects model, with 95% CIs. sAUC and pooled AUC (RE model) are based on different statistical assumptions and may yield different values. For studies without reported CIs for AUC, standard errors were approximated using the method of Hanley and McNeil (1982) for pooling [33].

^¶^ For AUC (RE model), one study (Sugimoto 2020) [Appendix S4] was excluded from the comparison vs. NHC, and another (Davies 2023) [17] from the comparison vs. ALS mimics because of not reporting AUC. Both studies were included in each Reitsma model (sAUC, summary sensitivity, and summary specificity).

^§^ One included study defined the control group by combining neurologically healthy and disease controls.

For blood biomarkers, the analysis included neurofilaments (NfL, pNfH, NfH) and GFAP. Most studies compared ALS with NHC, and among these, NfL demonstrated the highest performance (pooled sensitivity: 0.90, specificity: 0.91, SROC AUC: 0.96). In contrast, the meta‐analysis of studies comparing ALS with mimics was feasible only for NfL (pooled sensitivity: 0.83, specificity: 0.81, sAUC: 0.81), reflecting the scarcity of literature on other biomarkers (Figure 1a).

Regarding CSF biomarkers, the analysis included neurofilaments, chitinases (CHIT1, YKL40), tau proteins (t‐tau, p‐tau181), TDP‐43, and MCP1. Among these, CSF NfL exhibited the highest diagnostic value for distinguishing ALS from mimics (pooled sensitivity: 0.87, specificity: 0.86, sAUC: 0.92). CSF pNfH also showed a high diagnostic value (sAUC: 0.87). Chitinases and p‐tau/t‐tau ratio demonstrated moderate diagnostic accuracy (sAUC: 0.72–0.80), though based on a limited number of study cohorts (Figure 1b).

#### 3.2.2 Quality Assessment of Diagnostic Studies (QUADAS‐2)

All studies included in the SROC analyses were systematically assessed using the prespecified QUADAS‐2 protocol. QUADAS‐2 consists of four key components: (1) patient selection, (2) index test, (3) reference standard, and (4) flow and timing. The QUADAS‐2 assessment results for each study are provided in Figure S4.

Most studies (43/47) were rated as unclear or high risk of bias (RoB) in the patient selection domain, primarily due to retrospective, case–control designs. Reporting of patient selection procedures was often insufficient: only eight studies clearly stated whether enrollment was consecutive or random, and most did not provide a patient flow diagram.

Similarly, high RoB was observed in the index test domain. In 77% of the included studies (36/47), it was unclear whether biomarker testing was blinded to the reference standard (mostly clinical diagnostic criteria). Among the included studies, only Davies et al. [17] applied a prespecified cutoff for blood NfL in a prospective diagnostic accuracy study. Notably, the sensitivity and specificity reported in this study (0.77 and 0.75) were lower than those in other studies, implying that biases in patient selection and the optimized cutoffs may have contributed to the higher values reported elsewhere.

In contrast, the reference standard domain generally had a low RoB, as all studies consistently applied established clinical diagnostic criteria within each study. However, heterogeneity in the selection of diagnostic criteria (e.g., revised El Escorial, Awaji, and Gold Coast) across studies should be noted. Similarly, considerable variation was observed in the timing of biomarker measurements along the disease course. This reflects an inherent limitation of retrospective designs, where sampling may occur at variable intervals from symptom onset or diagnosis.

Overall, the quality of the included studies was limited. The systematic assessment revealed substantial variation in the reporting of methodological details across studies. As these limitations may lead to overestimation of diagnostic performance, the certainty of the pooled estimates should be considered low.

#### 3.2.3 Supplementary SMD Analyses

We conducted secondary analyses, pooling SMDs to evaluate the ability of biomarkers to distinguish ALS patients from controls. These analyses included 71 studies (ALS: 9025; HC: 4694; DC: 1373; mimics: 712). Summary results of the SMD analyses for all included biomarkers are presented in Table S1, with corresponding forest plots shown in Figures S5–S7.

The SMD analyses for blood biomarkers included neurofilaments, chitinases, GFAP, tau proteins, sEV‐TDP‐43, sTREM2, SPP1, UCHL1, creatinine, and metabolic hormone biomarkers (e.g., Leptin, Ghrelin, and C‐peptide). For comparisons against DCs and ALS mimics, the number of available studies was limited, except for NfL. When compared to NHCs, NfL exhibited the highest SMD, with a pooled estimate of 1.55 [1.34–1.75] (2028 ALS cases vs. 2420 controls), which aligns with findings from the primary SROC analysis. NfH, pNfH, and p‐tau181 showed a moderate SMD (0.87–1.10). Other biomarkers, including blood chitinases and GFAP, showed limited or inconsistent estimates. Although based on a single study with two independent cohorts, sEV‐TDP‐43 demonstrated a notably high SMD compared to NHC (1.53 [0.87–2.19]) and progressive supranuclear palsy (1.62 [1.25–1.99]), warranting further validation.

The SMD analyses for CSF biomarkers included neurofilaments, chitinases, GFAP, tau proteins, Aβ42, sTREM2, and ferritin. The high performance of neurofilaments and the moderate performance of chitinases were consistent in this analysis. Other biomarkers exhibited limited discriminatory ability. Notably, while p‐tau181 showed a moderate SMD in blood, no such elevation was observed in CSF.

### 3.3 Prognostic Values

For the prognostic analysis, 27 studies reporting HRs based on dichotomized biomarker values (e.g., above vs. below median) were included. Of these, 24 studies (5508 ALS patients) contributed to the meta‐analyses of multivariable Cox models, and 13 studies (2055 ALS patients) to those of univariable models.

#### 3.3.1 Survival Analyses: Pooled Cox Hazard Ratios

**FIGURE 2:**
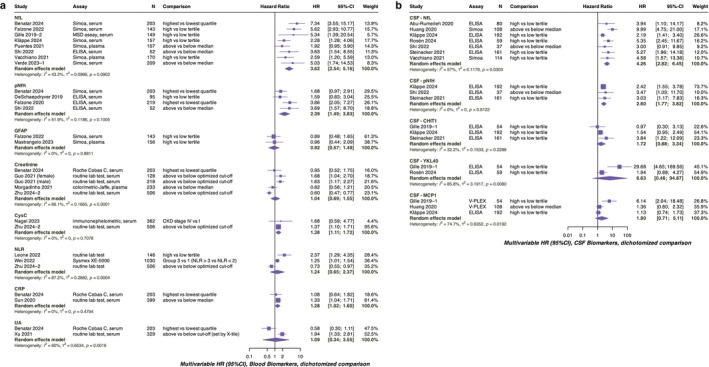
Prognostic Performance Based on Multivariable Cox Models (Blood and CSF Biomarkers). Panel (a) shows pooled hazard ratios for blood biomarkers, and panel (b) shows those for CSF biomarkers, both estimated using random‐effects models. All hazard ratios are derived from Cox proportional hazards models evaluating time to death or tracheostomy. The full list of included studies is provided in Appendix S4. ALS, amyotrophic lateral sclerosis; CHIT1, chitotriosidase 1; CSF, cerebrospinal fluid; CysC, cystatin C; GFAP, glial fibrillary acidic protein; MCP1, monocyte chemoattractant protein‐1; NfL, neurofilament light chain; NLR, neutrophil‐to‐lymphocyte ratio; pNfH, phosphorylated neurofilament heavy chain; UA, uric acid; YKL40, chitinase‐3‐like protein 1.

A summary of the pooled HR results for all included biomarkers is presented in Table 3, with the corresponding forest plots shown in Figure 2 (multivariable Cox model) and Figure S8 (univariable Cox model).

**TABLE 3:** Summary of pooled Cox hazard ratios.

Biomarker	Univariable Cox model			Multivariable Cox model		
	Cohorts	*N*	pooled HR [95% CI]	Cohorts	*N*	pooled HR [95% CI]
**Blood**						
NfL	5	765	4.09 [3.04; 5.49]	8	1190	3.62 [2.54; 5.16]
pNfH	2	147	2.52 [1.57; 4.06]	4	569	2.39 [1.49; 3.83]
GFAP	2	295	1.93 [0.98; 3.80]	2	299	0.92 [0.57; 1.49]
Creatinine	—	—	—	5	1288	1.04 [0.69; 1.55]
Cystatin C	—	—	—	2	868	1.38 [1.11; 1.72]
NLR	2	652	1.51 [0.38; 5.98]	3	1682	1.24 [0.65; 2.37]
CRP	—	—	—	2	602	1.28 [1.02; 1.60]
UA	—	—	—	2	532	1.09 [0.34; 3.55]
**CSF**						
NfL	3	309	2.75 [1.93; 3.92]	7	751	4.26 [2.82; 6.45]
pNfH	3	257	2.88 [1.66; 5.00]	3	390	2.60 [1.77; 3.82]
CHIT1	3	317	1.74 [1.05; 2.89]	3	407	1.72 [0.88; 3.34]
YKL40	—	—	—	2	113	6.63 [0.46; 94.87]
MCP1	2	297	1.79 [0.86; 3.72]	3	354	1.90 [0.71; 5.11]

For blood biomarkers (Figure 2a), the analysis included neurofilaments, GFAP, creatinine, cystatin C (CysC), C‐reactive protein (CRP), uric acid (UA), and neutrophil‐to‐lymphocyte ratio (NLR). Both NfL and pNfH demonstrated potential prognostic value in univariable and multivariable analyses, with NfL showing higher effect estimates (pooled multivariable HR: 3.62 [2.54–5.16] vs. pNfH HR: 2.39 [1.49–3.83]). In contrast, GFAP, creatinine, NLR, and UA showed no clear prognostic value. CysC and CRP showed modest associations with survival (HRs: 1.28–1.38), though these results were based on only two studies each.

The CSF biomarker analysis included neurofilaments, chitinases, and MCP1 (Figure 2b). Similarly to the diagnostic analyses, neurofilaments outperformed chitinases in prognostic value. In the multivariable pooled HR, chitinases and MCP1 demonstrated low certainty due to imprecision from small sample sizes. The pooled HRs of blood and CSF neurofilaments appear comparable, suggesting a potentially similar prognostic value; however, differences in study numbers and included covariates should be noted.

#### 3.3.2 Quality Assessment of Prognostic Studies (QUIPS)

For the quality assessment, all studies included in the pooled Cox HR analyses were evaluated using the prespecified QUIPS tool. QUIPS consists of six major domains: (1) study participation, (2) study attrition, (3) prognostic factor measurement, (4) outcome measurement, (5) study confounding, and (6) statistical analysis and reporting. An overview of the QUIPS assessment results is provided in Figure S9.

Regarding study participation, the number of eligible versus enrolled ALS patients was often missing. In several studies, the recruitment period and applied diagnostic criteria were unspecified, resulting in a moderate to high risk of bias (RoB). For study attrition, all studies were rated as moderate to high RoB, largely due to retrospective designs. Most did not report reasons for dropout, follow‐up completeness, or characteristics of participants lost to follow‐up. In contrast, most studies had low RoB for both prognostic factor measurement (26/27) and outcome measurement domains (24/27), as biomarkers were well defined and endpoints were objective (time to death or tracheostomy). However, several studies did not report follow‐up durations, making it unclear whether the observation period was sufficient to assess the outcome.

In the study confounding domain, most studies (24/27) included standard covariates and were rated as low RoB, though several concerns should be noted. First, while a similar set of covariates was often used, their combinations varied across studies (Table S2). Commonly adjusted covariates included age, sex, ALSFRS, disease duration (or diagnostic delay), baseline DPR, and site of onset; some studies also adjusted for vital capacity, genetic status, or frontotemporal dementia (FTD) status. Second, no study incorporated neurofilament levels as covariates in the multivariable models, leaving it unclear whether other biomarkers complement neurofilaments. Third, most studies did not specify how missing data were handled.

In the statistical analysis and reporting domain, moderate RoB was assigned to studies that relied on a single Cox model without justification or validation of covariate selection, due to the potential risk of selectively reporting significant results.

Overall, none of the included studies demonstrated uniformly low RoB across the six QUIPS domains. The heterogeneity in reporting and methodology limits the overall certainty in the pooled HR estimates, warranting cautious interpretation.

#### 3.3.3 Correlation with the Baseline DPR and ALS Functional Rating Scales

As supplementary analyses, we pooled Spearman's correlation coefficients between biomarkers and functional rating scales (ALSFRS and DPR). These analyses involved 58 studies, including 9945 ALS cases. Although correlations with these metrics do not directly indicate prognosis, both are established prognostic factors and are commonly included as covariates in multivariable Cox models. Table 4 summarizes the pooled correlation results for all included biomarkers, with corresponding forest plots shown in Figures S10 and S11.

**TABLE 4:** Summary of pooled correlation coefficients with functional measures.

Biomarker	Correlation with disease progression rate				Correlation with ALS Functional Rating Scales		
	Cohorts	N	Pooled rho [95% CI]		Cohorts	N	Pooled rho [95% CI]
**Blood**							
NfL	16	3126	0.51 [0.46; 0.55]		15	1602	−0.35 [−0.43; −0.27]
pNfH	5	356	0.45 [0.19; 0.71]		4	285	−0.42 [−0.70; −0.14]
NfH	3	217	0.25 [0.12; 0.39]		2	142	0.01 [−0.26; 0.28]
p‐tau181	—	—	—		4	478	−0.26 [−0.34; −0.17]
TDP‐43	—	—	—		2	234	0.06 [−0.07; 0.19]
sEV‐TDP‐43	—	—	—		2	230	−0.33 [−0.51; −0.15]
SPP1	—	—	—		2	228	−0.24 [−0.37; −0.12]
CK	4	1138	−0.12 [−0.18; −0.05]		3	916	0.26 [0.07; 0.44]
Creatinine	5	1262	−0.12 [−0.20; −0.04]		6	1617	0.33 [0.29; 0.37]
Cystatin C	—	—	—		2	1442	−0.17 [−0.22; −0.12]
IL‐2	2	299	−0.06 [−0.48; 0.37]		2	299	−0.01 [−0.56; 0.54]
IL‐6	2	327	0.22 [0.12; 0.32]		3	424	−0.25 [−0.34; −0.16]
IL‐10	2	327	0.17 [−0.15; 0.50]		—	—	—
TNF‐α	2	327	0.25 [0.07; 0.43]		—	—	—
**CSF**							
NfL	17	1519	0.45 [0.38; 0.52]		14	1256	−0.31 [−0.41; −0.22]
pNfH	11	738	0.48 [0.37; 0.60]		5	253	−0.41 [−0.63; −0.20]
NfH	2	178	0.37 [0.24; 0.50]		—	—	—
CHIT1	5	530	0.30 [0.20; 0.41]		4	563	−0.22 [−0.34; −0.10]
YKL40	3	230	0.28 [0.10; 0.46]		4	320	−0.25 [−0.44; −0.06]
t‐tau	2	272	0.14 [0.03; 0.26]		2	198	−0.09 [−0.24; 0.07]
p‐tau/t‐tau	2	277	−0.16 [−0.28; −0.05]		—	—	—
MCP1	2	229	0.24 [0.04; 0.44]		—	—	—
GFAP	—	—	—		2	195	−0.07 [−0.21; 0.07]

For blood biomarkers, the analysis included neurofilaments, creatinine, CysC, CK, interleukins, TNF‐α, p‐tau181, TDP‐43, sEV‐TDP‐43, and SPP1. Among these, NfL showed the strongest correlation with DPR (Spearman's ρ = 0.51 [95% CI: 0.46–0.55]), followed by pNfH. In contrast, inflammatory markers (IL‐6, IL‐10, and TNF‐α) demonstrated only weak correlations with DPR (ρ = 0.17–0.25). CK and creatinine were negatively correlated with DPR, albeit modestly (ρ = −0.12). Correlations with ALSFRS followed a similar pattern to those with DPR, but generally remained modest, with no biomarker showing a particularly high estimate.

For CSF biomarkers, the analysis included neurofilaments, chitinases (CHIT1 and YKL40), tau proteins (t‐tau and the p‐tau/t‐tau ratio), MCP1, and GFAP. Neurofilaments consistently showed the strongest correlation with DPR (e.g., CSF pNfH: ρ = 0.48 [0.37, 0.60]). In contrast, chitinases demonstrated moderate positive correlations (ρ = 0.28–0.32), the p‐tau/t‐tau ratio showed a weak negative correlation (ρ = −0.16), and others showed weaker or uncertain results. Regarding correlations with baseline ALSFRS, CSF NfL exhibited a moderate negative correlation (ρ = −0.31 [−0.41, −0.22]). Chitinases also showed weak negative correlations (ρ = −0.25 to −0.22), whereas t‐tau and GFAP did not demonstrate any significant association.

## 4 Discussion

In this systematic review and meta‐analysis, we evaluated 321 recent studies on diagnostic and prognostic biomarkers in ALS, focusing on blood and CSF. Among these, neurofilaments, particularly NfL, consistently demonstrated superior diagnostic and prognostic performance compared to other fluid biomarkers. To contextualize our findings, we first outline overall performance trends, then discuss key methodological limitations, and finally highlight critical knowledge gaps and directions for future research.

### 4.1 Diagnostic Biomarkers

Across diagnostic evaluations, NfL showed the most robust and consistent performance, while chitinases (CHIT1, YKL40) and the p‐tau/t‐tau ratio demonstrated moderate utility. The other biomarkers yielded limited or inconsistent results. Preliminary data from a single study also suggest promising diagnostic potential for sEV‐TDP‐43; however, further independent validation is necessary [21].

For most biomarkers, diagnostic performance in CSF and blood was generally concordant. A notable exception to this parallel CSF–blood pattern was p‐tau181. Several studies have reported elevated blood concentrations without a corresponding increase in CSF [34, 35]. A recent study, published after the end date of our systematic search, further supported this pattern, employing low‐molecular‐weight‐specific assays that are thought to detect p‐tau181 originating from both peripheral and central nervous systems [36]. These findings indicate that the increased plasma p‐tau181 levels may predominantly originate from peripheral sources. Further investigation into assay specificity and the biological source is warranted before its adoption as a reliable biomarker for ALS.

#### 4.1.1 Methodological Limitations of Diagnostic Studies

The QUADAS‐2 appraisal highlighted several key methodological limitations in current diagnostic biomarker studies. Most included studies employed retrospective case–control designs, often without a clearly defined patient enrollment. Diagnostic thresholds were rarely predetermined; instead, they were frequently established by optimizing the Youden index, with virtually no external validation and only rare internal validation (e.g., cross‐validation). These practices may result in overfitting and inflate estimates of diagnostic performance.

Moreover, substantial heterogeneity was observed across studies, including variability in applied diagnostic criteria (e.g., El Escorial, revised El Escorial, Awaji, Gold Coast), differences in included diagnostic certainty levels (suspected, possible, probable, definite), and inconsistent definitions of control groups. Given that distinguishing ALS from ALS mimics represents the most clinically relevant diagnostic challenge, the lower diagnostic performance observed in this comparison, along with the limited number of relevant studies, warrants particular attention. Indeed, several ALS mimics, including peripheral neuropathies, can also present with elevated neurofilament levels [17, 37, 38, 39, 40]. Consequently, setting an appropriate cutoff in this context becomes more challenging and requires greater stringency.

Another critical concern is the definition of ‘true ALS.’ Most studies relied solely on clinical diagnostic criteria at baseline, and only a few clarified whether all the included patients demonstrated disease progression after the initial evaluation. Given the risk of misclassification in the early stages [10, 11], clear reporting of disease progression is crucial to ensure the reliability of the diagnostic criteria as a reference standard. Systematic reporting of genetic status or neuropathological examination would provide even greater certainty, but such data have rarely been available. Consequently, the populations labeled as ‘ALS’ in these studies may have been heterogeneous and may have included patients with mimicking conditions. Therefore, reported diagnostic metrics should be interpreted as upper bounds rather than definitive values. Retrospective study designs further amplified this problem, as the same clinical criteria were often used both for patient inclusion and as the diagnostic reference standard. Such circularity obscures what the reported sensitivity and specificity truly represent and may inflate these estimates, particularly when information on longitudinal progression is lacking. Ideally, prospective cohorts should enroll a broad set of patients with suspected ALS at baseline and follow them over time to determine whether they exhibit typical progression. This would provide a more robust reference standard against which the diagnostic utility of baseline biomarkers could be assessed in a clinically more relevant context.

### 4.2 Prognostic Biomarkers

Across studies on prognosis, the performance hierarchy resembled that observed in the diagnostic analyses. Neurofilaments, particularly NfL, consistently showed the strongest associations with survival, whereas chitinases (CHIT1 and YKL40) provided moderate associations. Most other candidates offered limited or inconsistent prognostic value. The apparent overlap between diagnostic and prognostic performance may reflect the structure of current ALS criteria: diagnostic certainty increases as the disease advances, and later stages are inherently associated with shorter survival [41]. Among commonly measured systemic biomarkers, creatinine, UA, and NLR showed uncertain results, whereas CysC showed a modest association with survival; however, these findings are based on a limited number of studies and require further validation [42, 43].

#### 4.2.1 Methodological Limitations of Prognostic Studies

Several methodological issues limited the certainty of the pooled HRs. First, due to substantial heterogeneity in scaling of continuous biomarker values, we restricted the meta‐analysis to studies that reported Cox models based on dichotomized comparisons (e.g., above vs. below median). Although this approach avoids the influence of scaling, the absence of standardized cutoffs limited clinical interpretability. Second, covariate selection was inconsistent across studies, and sensitivity analyses regarding covariate selection were rarely performed. Some studies selected covariates using data‐driven procedures (e.g., stepwise approach) in modest sample sizes, which may have increased the risk of overfitting. In addition, closely related variables were sometimes included in the same model without assessing multicollinearity. For instance, several studies included both ALSFRS and DPR [44, 45, 46, 47] in the same model, despite DPR being derived directly from ALSFRS. Incorporating such highly correlated variables in the same model requires careful statistical consideration. Third, the handling of missing data was generally unspecified. None reported the use of multiple imputation methods or conducted sensitivity analyses using different assumptions for missing data. These issues highlight the need for standardized methodological guidelines, particularly regarding covariate selection and data transformation.

#### 4.2.2 Correlation With Functional Measures

In addition to the survival analyses, we examined the correlation between available biomarkers and functional rating scales (ALSFRS and baseline DPR). Neurofilaments and CSF chitinases showed moderate correlations with both measures, whereas most other biomarkers did not correlate with DPR but showed a weak correlation with ALSFRS. These findings suggest that many biomarkers correlate with disease burden at a single time point, but few capture the dynamics of disease progression. Additionally, longitudinal trajectories of the correlations between biomarkers and functional scales remain largely unexplored and warrant future research, particularly in the context of evaluating their utility as monitoring biomarkers.

### 4.3 Clinical Implications and Remaining Knowledge Gaps

Although NfL demonstrated the highest performance among the included biomarkers for ALS, it reflects general neuronal injury and has inherent limitations in both diagnostic and prognostic applications. There remains a great need for ALS pathology‐specific biomarkers, particularly to enhance differentiation from disease mimics that also cause neuronal injury.

**FIGURE 3:**
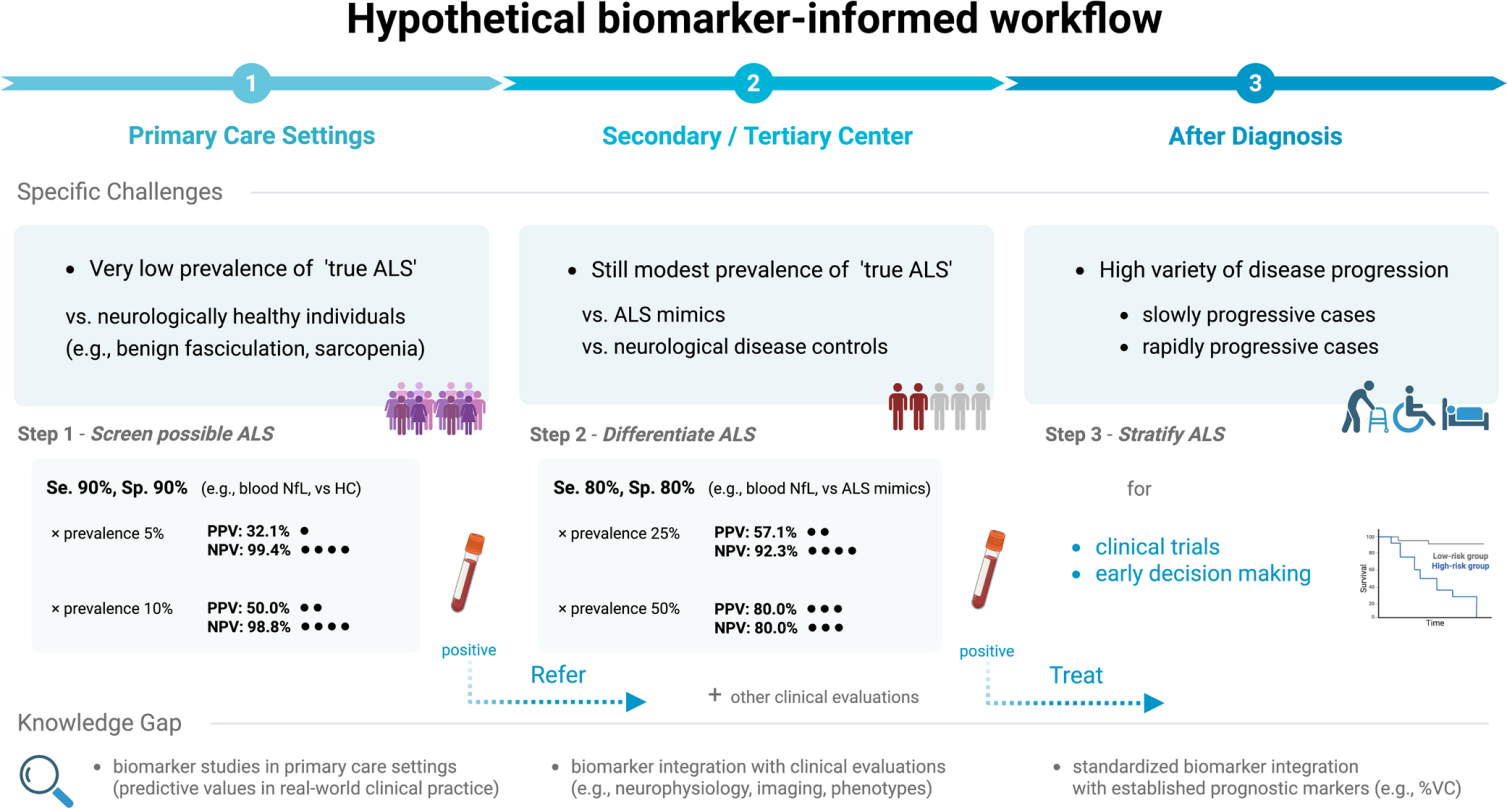
A Hypothetical Biomarker‐informed Workflow for ALS. The figure illustrates a three‐step clinical framework. In primary care settings (Step 1; assumed ALS prevalence 5%–10%), blood biomarkers with 90% sensitivity and specificity may help to rule out ALS, yielding high NPV. In secondary or tertiary care settings (Step 2; assumed prevalence ≥ 25%), their moderate PPV limits stand‐alone diagnostic use. After diagnosis (Step 3), biomarkers may support patient stratification by progression rate, though standardized prognostic models remain lacking. Dots indicate relative predictive values (visual aid only). ALS, amyotrophic lateral sclerosis; NfL, neurofilament light chain; NPV, negative predictive value; PPV, positive predictive value; Se., sensitivity; Sp., specificity; VC, vital capacity. Created with BioRender.com (Obara K. 2025).

To bridge the findings to clinical application, it is also crucial to consider how fluid biomarkers can be utilized across different stages of ALS care. As illustrated in Figure 3, fluid biomarkers may contribute at three distinct clinical stages: (1) as a screening tool in primary care, (2) aiding differential diagnosis in specialized clinics, and (3) stratifying disease progression after diagnosis.

In the diagnostic stage, clinical utility of biomarkers depends on predictive values, which are influenced not only by test sensitivity and specificity but also by the disease prevalence. In primary care settings (1), ALS prevalence is presumed to be very low. Assuming a hypothetical prevalence of 5%–10%, a biomarker with 90% sensitivity and specificity would yield a high negative predictive value (NPV≈99%). In this context, blood NfL, which increases even during the prediagnostic phase [48], may serve as a useful screening tool to support rule‐out or referral decision‐making for general practitioners. However, there remains a substantial gap in the literature regarding the optimal timing, method, and target population (e.g., individuals with benign fasciculations) for their implementation. Importantly, NfL alone cannot be used as a diagnostic tool in these settings, as the positive predictive value is very low (PPV≈32% at 5% prevalence, assuming 90% sensitivity and specificity).

In secondary and tertiary center settings (2), where ALS prevalence is assumed to be higher, the diagnostic challenges often involve distinguishing ALS from mimics. Based on our results, blood NfL offers only approximately 80% sensitivity and specificity in this context. Even under a simulated prevalence of 25% or 50%, this results in a modest (≈57%) to moderate PPV (≈80%), limiting its reliability as a stand‐alone diagnostic tool. These limitations underscore the need to integrate fluid biomarkers with other clinical evaluations to enhance diagnostic accuracy. Few studies have assessed whether combining fluid biomarkers with complementary modalities (e.g., imaging, neurophysiological tests, or additional biofluids) improves diagnostic accuracy [49]. Additionally, few studies explicitly accounted for concomitant cortical pathology or co‐existing FTD, which may confound biomarker levels by elevating them independently of motor neuron degeneration [50]. Incorporating these markers into diagnostic models may further improve the overall performance of diagnostic algorithms. In parallel, diagnostic performance may also be enhanced through the strategic implementation of biomarkers. A recent study on plasma p‐tau217 for AD introduced a two‐cutoff approach—one optimized for 95% sensitivity and the other for 95% specificity—which improved diagnostic accuracy across both primary and secondary care settings. This approach classified 12%–17% of individuals as intermediate, for whom further evaluation or confirmatory testing was recommended [51]. Similar approaches have not yet been explored in ALS biomarker research. Given its robust and consistent performance in our meta‐analysis, NfL could serve as the first step in a diagnostic algorithm. We suggest exploring two cutoff approaches for blood NfL to stratify individuals into low‐, high‐, and intermediate‐risk groups. For those in the intermediate (gray‐zone NfL values) group, complementary modalities such as CSF biomarkers, neurophysiological investigations, or imaging should be evaluated for the additional diagnostic value they may provide.

Following diagnosis (3), the major clinical need lies in stratifying patients by predicted disease progression speed, which would allow more efficient clinical trial design. This is particularly important when addressing clinical subtypes such as progressive muscular atrophy (PMA) [52]. PMA can be classified as ALS under the Gold Coast criteria but often follows a slower course distinct from typical ALS [9, 53]. This has raised debate over whether conditions with different progression patterns should be grouped together. Moreover, neurofilaments show only modest discriminatory ability when ALS and PMA are treated as dichotomous diagnostic labels [54, 55]. If baseline biomarkers can predict subsequent progression speed, patients could be stratified accordingly, addressing this heterogeneity without excluding PMA at the time of diagnosis. Notably, recent regulatory guidance has encouraged the use of prognostic covariates to adjust outcomes in clinical trials, further underscoring the importance of biomarker‐based prognostication [56]. Currently, however, standardized models incorporating fluid biomarkers with established prognostic factors (e.g., vital capacity) remain lacking. It also remains unclear whether other biomarkers offer independent or complementary prognostic value beyond NfL. Developing optimized multi‐modal biomarker models will require harmonized study designs that employ consistent diagnostic criteria, clearly defined control populations, standardized assay platforms, and prespecified cutoffs aligned with the clinical context.

In addition to informing prognosis, fluid biomarkers can support clinical trial design by serving as pharmacodynamic or response indicators. NfL has already been utilized as a surrogate outcome in trials [5]; however, it is crucial to carefully validate whether changes in biomarkers reliably predict clinically meaningful outcomes (e.g., survival) over the long term. Specifically, in this context, it is also essential to investigate how fluid biomarkers behave across different disease stages and genetic variants. To date, few studies have examined biomarker trajectories in ALS [57, 58], underscoring the need for further research. Future studies should also validate the timing and longitudinal stability of biomarker measurements to better define their role in diagnosis, prognosis, and patient monitoring. Other neurodegenerative diseases, such as AD, have substantially benefited from biomarker‐guided clinical trials, accelerating therapeutic development [12, 13]. ALS research could similarly progress through standardized biomarker study designs.

### 4.4 Strengths and Limitations

A major strength of this review is its comprehensive coverage of recent studies on various candidate biomarkers. By systematically synthesizing diagnostic and prognostic performance across multiple biomarkers, this review provides a comparative overview that offers insights into their clinical utility and guides future research.

Several limitations must also be acknowledged. First, restricting the search to studies published from 2019 onward excluded earlier investigations. We adopted this time window to ensure relevance to contemporary research and clinical practice, focusing on biomarkers measured with modern platforms (e.g., Simoa and Ella) [34, 40, 44, 47, 58, 59, 60], and on studies applying the recent diagnostic criteria [8, 10, 17, 59]. Despite this narrower window, our results align with those of earlier systematic reviews [23, 24, 25, 26, 27, 28, 61, 62]. Second, substantial methodological heterogeneity limited the certainty of pooled estimates and complicated head‐to‐head comparisons. Third, the minimum eligible sample size was set at 10 to avoid omitting relevant studies; this carried the theoretical risk that pooling multiple very small cohorts could bias the estimates, particularly when such studies predominated. In practice, however, their contribution to the total weight was limited, and the pooled results were not materially affected by their exclusion. Finally, the assessment of publication bias was feasible only for NfL, the sole biomarker with more than 10 eligible studies. Funnel‐plot symmetry and Egger's test did not indicate reporting bias for NfL, but the possibility of unreported negative findings cannot be dismissed for the other biomarkers [32]. Until larger, prospectively registered studies become available, concerns about selective reporting should be considered when interpreting the current literature.

## 5 Conclusions

Among fluid biomarkers, NfL exhibited the highest diagnostic and prognostic value, both in blood and CSF. However, its diagnostic accuracy is limited when used alone, particularly in low‐prevalence settings. Moderate utility was observed for chitinases and the p‐tau/t‐tau ratio, while other biomarkers demonstrated limited or inconsistent results. Most included studies had moderate to high risks of bias, and the overall certainty of the reported effect estimates was low. Prospective studies with standardized protocols and external validation are essential to confirm these findings and to advance biomarker‐guided clinical practice.

## Author Contributions

K.O.: conceptualization, methodology, formal analysis, investigation, data curation, writing – original draft, writing – review and editing, visualization, resources, project administration, and funding acquisition. D.I.: investigation, data curation, and writing – review and editing. C.N.: writing – review and editing, supervision. A.S.: writing – review and editing, supervision. S.J.: writing – review and editing, supervision. M.K.: investigation, writing – review and editing, and supervision. N.M.‐C.: conceptualization, methodology, writing – original draft, writing – review and editing, project administration, supervision, and funding acquisition.

## Conflicts of Interest

The authors declare that the research was conducted in the absence of any commercial or financial relationships that could be construed as potential conflicts of interest. N.M.‐C. has received speaker and consultancy fees from BioArctic, Biogen, Eli Lilly, Owkin, and Merck, which are not related to the present work.

## Supporting information


**Appendix S1:** Search Report.

**Appendix S2:** The Protocol of QUADAS‐2 for ALS Biomarker Systematic Review.

**Appendix S3:** QUIPS tool Protocol for the ALS Biomarker Systematic Review and Meta‐Analysis.

**Appendix S4:** ene70382‐sup‐0004‐Appendix4.xlsx.

**Figure S1:** PRISMA Flow Diagram of Study Selection.
The diagram outlines the number of records identified, screened, assessed for eligibility, and included in the final analysis, following the PRISMA 2020 guidelines.

**Figure S2:** Pooled AUCs for Differentiating ALS from NHC (Blood and CSF Biomarkers). Panel (a) shows pooled areas under the receiver operating characteristic curves (AUCs) for blood biomarkers, and panel (b) shows those for CSF biomarkers, both estimated using random‐effects models.

**Figure S3:** Pooled AUCs for Differentiating ALS from DC/ALS Mimics (Blood and CSF Biomarkers). Panel (a) shows pooled areas under the receiver operating characteristic curves (AUCs) for blood biomarkers, and panel (b) shows those for CSF biomarkers, both estimated using random‐effects models.

**Figure S4:** Overview of QUADAS‐2 Assessment. The figure summarizes domain‐level judgments based on consensus between two independent reviewers, according to the QUADAS‐2 framework. QUADAS‐2, Quality Assessment of Diagnostic Accuracy Studies 2.

**Figure S5:** Pooled SMDs for ALS vs. ALS Mimics (Blood and CSF Biomarkers). Panel (a) shows pooled Standardized Mean Differences (SMDs) for blood biomarkers, and panel (b) shows those for CSF biomarkers, both estimated using random‐effects models.

**Figure S6:** Pooled SMDs for ALS vs. NHC (Blood and CSF Biomarkers).
Panel (a) shows pooled Standardized Mean Differences (SMDs) for blood biomarkers, and panel (b) shows those for CSF biomarkers, both estimated using random‐effects models.

**Figure S7:** Pooled SMDs for ALS vs. DC/ALS Mimics (Blood and CSF Biomarkers). Panel (a) shows pooled Standardized Mean Differences (SMDs) for blood biomarkers, and panel (b) shows those for CSF biomarkers, both estimated using random‐effects models.

**Figure S8:** Forest Plots of Pooled Univariable Cox Hazard Ratios (Blood and CSF Biomarkers). Panel (a) shows pooled hazard ratios (HRs) for blood biomarkers, and panel (b) shows those for CSF biomarkers, both estimated using random‐effects models. All HRs are derived from Cox proportional hazards models evaluating time to death or tracheostomy.

**Figure S9:** Overview of QUIPS assessment. The figure summarizes domain‐level judgments based on consensus between two independent reviewers, according to the QUIPS framework. QUIPS, quality in prognosis studies.

**Figure S10:** Pooled Correlation Coefficients Between Blood/CSF Biomarkers and DPR. Panel (a) shows pooled correlation coefficients for blood biomarkers, and panel (b) shows those for CSF biomarkers, both estimated using random‐effects models.

**Figure S11:** Pooled Correlation Coefficients Between Blood/CSF Biomarkers and ALSFRS. Panel (a) shows pooled correlation coefficients for blood biomarkers, and panel (b) shows those for CSF biomarkers, both estimated using random‐effects models.

**Table S1:** Summary of pooled standardized mean differences (SMDs).
**Table S2:** Covariates included in multivariable Cox regression models.

## Data Availability

All datasets generated and/or analyzed during this study are provided in Appendix S4. All analysis scripts used in this study are available at https://github.com/obkz/ALS_biomarker_SRMA.
