# Supplementary material for: Diagnostic and Prognostic Value of Blood and Cerebrospinal Fluid Biomarkers in Amyotrophic Lateral Sclerosis: A Systematic Review and Meta‐Analysis
Source: Eur J Neurol. 2025 Oct 27;32(10):e70382. doi: 10.1111/ene.70382 (PMC12554952; doi:10.1111/ene.70382)
Supplement: Supplementary file 1 — Appendix S1: Search Report. [file ENE-32-e70382-s004.pdf]

Krister Aronsson, Librarian / Information specialist
Library & ICT, Faculty of Medicine
Lund University
Krister.aronsson@med.lu.se

SEARCH REPORT

PROJECT INFORMATION

REQUEST DATE	NAME	INSTITUTION/ORGANISATION
20240618	Kazuki Obara<br>Niklas Mattsson-Carlgren	Faculty of medicine

PROJECT TIME FRAME – DESIRED DATE OF DELIVERY

20240805

DESCRIBE RESEARCH QUESTION AND PURPOSE WITH THE SEARCH

Recent advancements in Cerebrospinal Fluid (CSF)/plasma biomarkers for the diagnosis, prognosis, and monitoring of neurological diseases: A systematic review and meta-analysis

Prognosis:
How do fluid (CSF/plasma) biomarkers correlate with prognosis, such as ALSFRS-R slope and time to death/intubation in patients with ALS?

Diagnosis:
What is the sensitivity and specificity of fluid (CSF/plasma) biomarkers in diagnosing Amyotrophic Lateral Sclerosis (ALS)?

STRUCTURED RESEARCH QUESTION

PROVIDE THE RESEARCH QUESTION IN A STURCTURED FORMAT
:PICO(S), PEO OR IN FREE ORDER

Patient / population / problem	Population

Intervention	Exposure
Comparison / control	Outcome
Outcome	
Studytype(s)	

REFERENCES TO KEY ARTICLES – EXAMPLES OF YOU WANT TO HAVE INCLUDED IN THE SEARCH RESULT (3-5 ARTICLES)

Diagnosis:

Agnello L, Colletti T, Lo Sasso B, et al. Tau protein as a diagnostic and prognostic biomarker in amyotrophic lateral sclerosis. Eur J Neurol. 2021;28(6):1868-1875. doi:10.1111/ene.14789
https://pubmed.ncbi.nlm.nih.gov/33638255/

Cousins KAQ, Shaw LM, Shellikeri S, et al. Elevated Plasma Phosphorylated Tau 181 in Amyotrophic Lateral Sclerosis. Ann Neurol. 2022;92(5):807-818. doi:10.1002/ana.26462
https://pubmed.ncbi.nlm.nih.gov/35877814/

Thompson AG, Gray E, Bampton A, Raciborska D, Talbot K, Turner MR. CSF chitinase proteins in amyotrophic lateral sclerosis. J Neurol Neurosurg Psychiatry. 2019;90(11):1215-1220. doi:10.1136/jnnp-2019-320442
https://pubmed.ncbi.nlm.nih.gov/31123140/

Prognosis

Benatar M, Zhang L, Wang L, et al. Validation of serum neurofilaments as prognostic and potential pharmacodynamic biomarkers for ALS. Neurology. 2020;95(1):e59-e69. doi:10.1212/WNL.0000000000009559
https://pubmed.ncbi.nlm.nih.gov/32385188/

Ingre C, Chen L, Zhan Y, Termorshuizen J, Yin L, Fang F. Lipids, apolipoproteins, and prognosis of amyotrophic lateral sclerosis. Neurology. 2020;94(17):e1835-e1844. doi:10.1212/WNL.0000000000009322
https://pubmed.ncbi.nlm.nih.gov/32221024/

Thompson AG, Gray E, Thézénas ML, et al. Cerebrospinal fluid macrophage biomarkers in amyotrophic lateral sclerosis. Ann Neurol. 2018;83(2):258-268. doi:10.1002/ana.25143
https://pubmed.ncbi.nlm.nih.gov/29331073/

DESIRED DELIVERY FORMAT (ENDNOTE, WORD, PDF, COVIDENCE)

Endnote

SEARCH STRATEGIES

The search strategy documentation is to be cited as below, for protocol or publication in journal as appendix. The documentation is structured according to PRISMA 2020, international standard for systematic reviews. Please note that the information specialist delivers the parts required for search strategy documentation, additional method information may also be required.

See PRISMA 2020 for further information:
http://prisma-statement.org/
http://prisma-statement.org/Extensions/Searching

Databases

PubMed (National Library of Medicine, 1946-present)
Date of search:20240722
#1
"Amyotrophic Lateral Sclerosis"[Mesh]
=24616 records

#2
Amyotrophic lateral sclerosis[Title/Abstract] OR ALS[Title/Abstract] OR gehrig disease[Title/Abstract] OR gehrig s disease[Title/Abstract] OR gehrig's disease[Title/Abstract] OR guam disease[Title/Abstract]
=40980 records

#3
#1 OR #2
=43364 records

#4
((("Cerebrospinal Fluid"[Mesh]) OR "Plasma"[Mesh]) OR "Serum"[Mesh]) OR "Blood"[Mesh]
=1231894 records

#5
Cerebrospinal fluid* OR CSF OR plasma* OR blood* OR serum*
=5585446 records

#6
#4 OR #5
=626556 records

#7
"Biomarkers"[Mesh]
=912168 records

#8
Biomarker* OR tau OR chitinase protein OR biochemical marker* OR biological marker*
=1277294 records

#9
#7 OR #8
=1541579 records

Prognosis

#10
Progression OR slope OR ALSFRS OR survival OR elevated OR elevation OR progress
=4844951 records

#11
#3 AND #6 AND #9 AND #10
=907 records

#12
#11 Filters: from 2014 – 2024
=**688 records**

Diagnosis

#13
diagnos*
=6396191 records

#14
sensitivity OR predictive value OR specificity OR AUC
=6220105 records

#15
#3 AND #6 AND #9 AND #13 AND #14
=365 records

#16
#15 Filters: from 2014-2024
=**280 records**

Total
#12/#16
=**763 records**

Embase.com (Elsevier, 1947-present)

Date of search: 20240722
#1
'amyotrophic lateral sclerosis'/exp OR 'amyotrophic lateral sclerosis':ti,ab,kw OR als:ti,ab,kw OR 'gehrig disease':ti,ab,kw OR 'gehrigs disease':ti,ab,kw OR 'guam disease':ti,ab,kw
=73790 records

#2
'cerebrospinal fluid'/exp OR 'blood'/exp OR 'plasma'/exp OR 'serum'/exp OR (cerebrospinal AND fluid*) OR csf OR plasma* OR blood* OR serum*
=8948057 records

#3
'biological marker'/exp OR ((((biomarker* OR tau OR chitinase) AND protein OR biochemical) AND marker* OR biological) AND marker*)
=728810 records

Prognosis

#4

progression OR slope OR alsfrs OR survival OR elevated OR elevation OR progress
=4778617 records

#5
#1 AND #2 AND #3 AND #4
=1022 records

#6
#5 AND (2014:py OR 2015:py OR 2016:py OR 2017:py OR 2018:py OR 2019:py OR 2020:py OR 2021:py OR 2022:py OR 2023:py OR 2024:py) AND [embase]/lim NOT ([embase]/lim AND [medline]/lim)
=**478 records**

Diagnosis

#7
diagnos* AND ('sensitivity'/exp OR sensitivity OR 'predictive value'/exp OR 'predictive value' OR (predictive AND ('value'/exp OR value)) OR 'specificity'/exp OR specificity OR 'auc'/exp OR auc)
=950178 records

#8
#1 AND #2 AND #3 AND #4
=295 records

#9
#(2014:py OR 2015:py OR 2016:py OR 2017:py OR 2018:py OR 2019:py OR 2020:py OR 2021:py OR 2022:py OR 2023:py OR 2024:py) AND [embase]/lim NOT ([embase]/lim AND [medline]/lim)
=101 records

Total
#6/#9
=523 records

CINAHLComplete (Cumulative Index to Nursing and Allied Health Literature; EbscoHost, inception to present)

Date of search:20240723
#1
(MM "Amyotrophic Lateral Sclerosis") OR TI (Amyotrophic lateral sclerosis OR ALS OR gehrig disease OR gehrigs disease OR gehrig’s disease OR guam disease) OR AB (Amyotrophic lateral sclerosis OR ALS OR gehrig disease OR gehrigs disease OR gehrig’s disease OR guam disease)
=11374 records

#2
((MM "Cerebrospinal Fluid") OR (MH "Blood+")) OR (Cerebrospinal fluid* OR CSF OR plasma* OR blood* OR serum*)
=751956 records

#3
(MH "Biological Markers+") OR (Biomarker* OR tau OR chitinase protein OR biochemical marker* OR biological marker*)
=167149 records

Prognostics

#4
Progression OR slope OR ALSFRS OR survival OR elevated OR elevation OR progress
=525059 records

#5
#1 AND #2 AND #3 AND #4
=100 records

#6
#5
Limiters - Publication Date: 20140101-20241231
=**79 records**

Diagnostics

#7
diagnos*
=1256438 records

#8
sensitivity OR predictive value OR specificity OR AUC
=312890 records

#9
#1 AND #2 AND #3 AND #7 AND #8
=35 records

#10
#9 Limiters - Publication Date: 20140101-20241231
=**26 records**

Total: **90 records**

Cochrane Library via Cochrane Library Online (Wiley, Issue 2 of 12, February 2022)

Date of search:20240722
#1
MeSH descriptor: [Amyotrophic Lateral Sclerosis] explode all trees
=898 records

#2
(Amyotrophic lateral sclerosis OR ALS OR gehrig disease OR gehrigs disease OR gehrig's disease OR guam disease):ti,ab,kw
=40825 records

#3
#1 OR #2
=40825 records

#4
MeSH descriptor: [Cerebrospinal Fluid] explode all trees
=163 records

#5

MeSH descriptor: [Blood] explode all trees
=21253 records

#6
MeSH descriptor: [Plasma] explode all trees
=1979 records

#7
MeSH descriptor: [Serum] explode all trees
=1077 records

#8
(Cerebrospinal fluid* OR CSF OR plasma* OR blood* OR serum*):ti,ab,kw
=544999 records

#9
#4 OR #5 OR #6 OR #7 OR #9
=548947 records

#10
MeSH descriptor: [Biomarkers] explode all trees
=31597 records

#11
(Biomarker* OR tau OR chitinase protein OR biochemical marker* OR biological marker*):ti,ab,kw
=71272 records

#12
#10 OR #11
=77764 records

Prognosis

#13
(Progression OR slope OR ALSFRS OR survival OR elevated OR elevation OR progress):ti,ab,kw
=277923 records

#14
#3 AND #9 AND #12 AND #13
=690 records

#15
with Cochrane Library publication date from Jan 2014 to Dec 2024
=**668 records (all trials)**

Diagnosis

#16
(diagnos*):ti,ab,kw
=333199 records

#17
(sensitivity OR predictive value OR specificity OR AUC):ti,ab,kw
=284157 records*

#18
#3 AND #9 AND #12 AND #16 AND #17
=232 records

#19
#18 with Cochrane Library publication date from Jan 2014 to Dec 2024
=**223 records (all trials)**

Total:
#15/#19
=**757 records**

PsycInfo (EbscoHost, inception to present)

Date of search: 20240723
#1
MM "Amyotrophic Lateral Sclerosis" OR TI (Amyotrophic lateral sclerosis OR ALS OR gehrig disease OR gehrigs disease OR gehrig’s disease OR guam disease) OR AB (Amyotrophic lateral sclerosis OR ALS OR gehrig disease OR gehrigs disease OR gehrig’s disease OR guam disease)
=18401 records

#2
((DE "Cerebrospinal Fluid") OR (DE "Blood" OR DE "Blood Plasma")) OR (Cerebrospinal fluid* OR CSF OR plasma* OR blood* OR serum*)
=169616 records

#3
(DE "Biological Markers" OR DE "Inflammatory Markers") OR (Biomarker* OR tau OR chitinase protein OR biochemical marker* OR biological marker*)
=60377 records

Prognostics

#4
Progression OR slope OR ALSFRS OR survival OR elevated OR elevation OR progress
=263990 records

#5
#1 AND #2 AND #3 AND #4
=128 records

#6
#5 Limiters - Publication Year: 2014-2024
=**85 records**

Diagnostics

#7
diagnos*
=449332 records
#8
diagnos*
=449932 records

#9
#1 AND #2 AND #3 AND #7 AND #8
=57 records

#10
#9 Limiters - Publication Year: 2014-2024
=**39 records**

Total: 85 records

Web of Science Core collection (Clarivate Analytics)
Date of search: 20240723
#1
TS=(Amyotrophic lateral sclerosis OR ALS OR gehrig disease OR gehrigs disease OR gehrig's disease OR guam disease)
=70525 records

#2
ALL=(Cerebrospinal fluid* OR CSF OR plasma* OR blood* OR serum*)
=5976266

#3
ALL=(Biomarker* OR tau OR chitinase protein OR biochemical marker* OR biological marker*)
=921348

Prognostics

#4
ALL=(Progression OR slope OR ALSFRS OR survival OR elevated OR elevation OR progress)
=4834836 records

#5
#1 AND #2 AND #3 AND #4
=910 records

#6

#1 AND #2 AND #3 AND #4 and 2014 or 2015 or 2016 or 2017 or 2018 or 2019 or 2020 or 2021 or 2022 or 2023 or 2024 (Publication Years)
=**743 records**

Diagnostics
#7
ALL=(diagnos*)
=3926944 records

#8
ALL=(sensitivity OR predictive value OR specificity OR AUC)
=2559983 records

#9
#1 AND #2 AND #3 AND #7 AND #8
=184 records

#10
#1 AND #2 AND #3 AND #7 AND #8 and 2014 or 2015 or 2016 or 2017 or 2018 or 2019 or 2020 or 2021 or 2022 or 2023 or 2024 (Publication Years)
=154 records

Databases searched in Web of Science Core Collection (inception as described below)
Science Citation Index Expanded (SCI-EXPANDED) --1900-present
Social Sciences Citation Index (SSCI) --1956-present
Arts & Humanities Citation Index (A&HCI) --1975-present
Conference Proceedings Citation Index- Science (CPCI-S) --1990-present
Conference Proceedings Citation Index- Social Science & Humanities (CPCI-SSH) --1990-present
Emerging Sources Citation Index (ESCI) --2015-present

Total number of records from databases:

Prognosis:
Before deduplication: 2714 records
After deduplication: 2110 records
Publication from 2019-01-01: 1246 (864 removed), filtered by KO

Diagnosis:
Before deduplication: 823 records
After deduplication: 686 records
Publication from 2019-01-01: 401 (285 removed), filtered by KO

Total:
2796 records
Publication from 2019-01-01: 1647 (1149 removed), filtered by KO

Complementary search
for articles 2024-07-01-to present
Date of search: 20250324-20250325

PubMed (National Library of Medicine, 1946-present)
Date of search:20250324
#1
"Amyotrophic Lateral Sclerosis"[Mesh]
=24415 records

#2
Amyotrophic lateral sclerosis[Title/Abstract] OR ALS[Title/Abstract] OR gehrig disease[Title/Abstract] OR gehrig s disease[Title/Abstract] OR gehrig's disease[Title/Abstract] OR guam disease[Title/Abstract]
=43614 records

#3
#1 OR #2
=46005 records

#4
((("Cerebrospinal Fluid"[Mesh]) OR "Plasma"[Mesh]) OR "Serum"[Mesh]) OR "Blood"[Mesh]
=1248278 records

#5
Cerebrospinal fluid* OR CSF OR plasma* OR blood* OR serum*
=5721332 records

#6
#4 OR #5
=6270390 records

#7
"Biomarkers"[Mesh]
=941559 records

#8
Biomarker* OR tau OR chitinase protein OR biochemical marker* OR biological marker*
=1342780 records

#9
#7 OR #8
=1611335 records

#10
("2024/07/01"[Date - Publication] : "3000"[Date - Publication])
=1292697 records

Prognosis

#11
Progression OR slope OR ALSFRS OR survival OR elevated OR elevation OR progress
=5050151 records

#12
#3 AND #6 AND #9 AND #10 AND #11
=**107 records**

Diagnosis

#13
diagnos*
=6619714 records

#14
sensitivity OR predictive value OR specificity OR AUC
=6470941 records

#15
#3 AND #6 AND #9 AND #10 AND #13 AND #14
=**38 records**

Total

#16
#12 OR #15
=**115 records**

Embase.com (Elsevier, 1947-present)
Date of search: 20250324
#1
'amyotrophic lateral sclerosis'/exp OR 'amyotrophic lateral sclerosis':ti,ab,kw OR als:ti,ab,kw OR 'gehrig disease':ti,ab,kw OR 'gehrigs disease':ti,ab,kw OR 'guam disease':ti,ab,kw
=76599 records

#2
'cerebrospinal fluid'/exp OR 'blood'/exp OR 'plasma'/exp OR 'serum'/exp OR (cerebrospinal AND fluid*) OR csf OR plasma* OR blood* OR serum*
=9221283 records

#3
'biological marker'/exp OR ((((biomarker* OR tau OR chitinase) AND protein OR biochemical) AND marker* OR biological) AND marker*)
=783317 records

#4
#1 AND #2 AND #3
=2044 records

#5

#5
#4 #1 AND #2 AND #3 AND [01-07-2024]/sd NOT [01-04-2025]/sd
=172 records

Prognosis

#6
progression OR slope OR alsfrs OR survival OR elevated OR elevation OR progress
=4994321 records

#7

#4 AND #5
=100 records

#8
diagnos* AND ('sensitivity'/exp OR sensitivity OR 'predictive value'/exp OR 'predictive value' OR (predictive AND ('value'/exp OR value)) OR 'specificity'/exp OR specificity OR 'auc'/exp OR auc)
=1006540 records

#9
#4 AND #8
=35 records

Total

#10
#7 OR #9
=**116 records**

CINAHLComplete (Cumulative Index to Nursing and Allied Health Literature; EbscoHost, inception to present)
Date of search:20250324
#1
(MM "Amyotrophic Lateral Sclerosis") OR TI (Amyotrophic lateral sclerosis OR ALS OR gehrig disease OR gehrigs disease OR gehrig’s disease OR guam disease) OR AB (Amyotrophic lateral sclerosis OR ALS OR gehrig disease OR gehrigs disease OR gehrig’s disease OR guam disease)
=11754 records

#2
((MM "Cerebrospinal Fluid") OR (MH "Blood+")) OR (Cerebrospinal fluid* OR CSF OR plasma* OR blood* OR serum*)
=765752 records

#3
(MH "Biological Markers+") OR (Biomarker* OR tau OR chitinase protein OR biochemical marker* OR biological marker*)
=172426 records

#4
#1 AND #2 AND #3
=181 records

#5
#4 Limiters - Publication Date: 20240701-20250331
=**6 records**

Prognostics

#6
Progression OR slope OR ALSFRS OR survival OR elevated OR elevation OR progress

=536285 records

#7
#5 AND #6
=**2 records**

Diagnostics

#8
diagnos*
=1279053 records

#9
sensitivity OR predictive value OR specificity OR AUC
=319856 records

#10
#5 AND #8 AND #9
=**2 records**

Total

#11
#7 OR #10
=**3 records**

the Cochrane Central Register of Controlled Trials (CENTRAL; 2023; Issue 8) in the Cochrane Library
Date of search:20250324
#1
MeSH descriptor: [Amyotrophic Lateral Sclerosis] explode all trees
=892 records

#2
(Amyotrophic lateral sclerosis OR ALS OR gehrig disease OR gehrigs disease OR gehrig's disease OR guam disease):ti,ab,kw
=43793 records

#3
#1 OR #2
=40825 records

#4
MeSH descriptor: [Cerebrospinal Fluid] explode all trees
=164 records

#5
MeSH descriptor: [Blood] explode all trees

=20850 records

#6
MeSH descriptor: [Plasma] explode all trees
=1965 records

#7
MeSH descriptor: [Serum] explode all trees
=1055 records

#8
(Cerebrospinal fluid* OR CSF OR plasma* OR blood* OR serum*):ti,ab,kw
=553868 records

#9
#4 OR #5 OR #6 OR #7 OR #9
=557756 records

#10
MeSH descriptor: [Biomarkers] explode all trees
=31226 records

#11
(Biomarker* OR tau OR chitinase protein OR biochemical marker* OR biological marker*):ti,ab,kw
=73041 records

#12
#10 OR #11
=79519 records

#13
#3 AND #9 AND #12
=1437 records

#14
#13
with Cochrane Library publication date from Jul 2024 to Mar 2025
=105 records

Prognosis

#15
(Progression OR slope OR ALSFRS OR survival OR elevated OR elevation OR progress):ti,ab,kw
=284018 records

#16
#14 AND #15
=**56 records**

Diagnosis

#17
(diagnos*):ti,ab,kw
=341784 records

#18
(sensitivity OR predictive value OR specificity OR AUC):ti,ab,kw
=290445 records

#19
#14 AND #17 AND #18
=**13 records**

Total

#20
#16 OR #19
=**61 records, all trials**

PsycInfo (EbscoHost, inception to present)
Date of search: 20240325
#1
MM "Amyotrophic Lateral Sclerosis" OR TI (Amyotrophic lateral sclerosis OR ALS OR gehrig disease OR gehrigs disease OR gehrig's disease OR guam disease) OR AB (Amyotrophic lateral sclerosis OR ALS OR gehrig disease OR gehrigs disease OR gehrig's disease OR guam disease)
=19103 records

#2
((DE "Cerebrospinal Fluid") OR (DE "Blood" OR DE "Blood Plasma")) OR (Cerebrospinal fluid* OR CSF OR plasma* OR blood* OR serum*)
=173320 records

#3
(DE "Biological Markers" OR DE "Inflammatory Markers") OR (Biomarker* OR tau OR chitinase protein OR biochemical marker* OR biological marker*)
=63565 records

#4
#1 AND #2 AND #3
=265 records

#5
#4
Limiters - Publication Year: 2024-2025
=**25 records**

Prognostics

#6
Progression OR slope OR ALSFRS OR survival OR elevated OR elevation OR progress
=271571 records

#7
#5 AND #6
=**13 records**

Diagnostics

#8
diagnos*
=461014 records

#9
sensitivity OR predictive value OR specificity OR AUC
=204848 records

#10
#5 AND #8 AND #9
=**3 records**

Total:

#11
=14 records

Web of Science Core collection (Clarivate Analytics)
Date of search: 20250325
#1
TS=(Amyotrophic lateral sclerosis OR ALS OR gehrig disease OR gehrigs disease OR gehrig's disease OR guam disease)
=75831 records

#2
ALL=(Cerebrospinal fluid* OR CSF OR plasma* OR blood* OR serum*)
=6311976 records

#3
ALL=(Biomarker* OR tau OR chitinase protein OR biochemical marker* OR biological marker*)
=1009464 records

#4
#1 AND #2 AND #3
=1855 records

#5
#4 and 2026 or 2025 or 2024 (Publication Years)
=**230 records**

Prognostics

#6
ALL=(Progression OR slope OR ALSFRS OR survival OR elevated OR elevation OR progress)
=5267562 records

#7
#5 AND #6
=**140 records**

Diagnostics

#8
ALL=(diagnos*)
=4369142 records

#9
ALL=(sensitivity OR predictive value OR specificity OR AUC)
=2765165 records

#10
#5 AND #8 AND #9
=**23 records**

Total

#11
#7 OR #10
=**147 records**

Databases searched in Web of Science Core Collection (inception as described below)
Science Citation Index Expanded (SCI-EXPANDED) --1900-present
Social Sciences Citation Index (SSCI) --1956-present
Arts & Humanities Citation Index (A&HCI) --1975-present
Conference Proceedings Citation Index- Science (CPCI-S) --1990-present
Conference Proceedings Citation Index- Social Science & Humanities (CPCI-SSH) --1990-present
Emerging Sources Citation Index (ESCI) --2015-present

Total number of records from databases (for articles 2024-07-01-to present):

Before deduplication: 456 records
After deduplication: 269 records
