# Supplementary material for: Diagnostic and Prognostic Value of Blood and Cerebrospinal Fluid Biomarkers in Amyotrophic Lateral Sclerosis: A Systematic Review and Meta‐Analysis
Source: Eur J Neurol. 2025 Oct 27;32(10):e70382. doi: 10.1111/ene.70382 (PMC12554952; doi:10.1111/ene.70382)
Supplement: Supplementary file 2 — Appendix S2: The Protocol of QUADAS‐2 for ALS Biomarker Systematic Review. [file ENE-32-e70382-s013.docx]

# **The Protocol of QUADAS-2 for ALS biomarker Systematic Review**

This document outlines the prespecified protocol for assessing risk of bias and applicability concerns using the QUADAS-2, as originally developed by Whiting et al. (*Ann Intern Med. 2011;155(8):529–536*).

An initial version was drafted by KO and DI prior to completion of data extraction and finalized after discussion. The finalized protocol was then applied consistently to all diagnostic studies included in the summary ROC meta-analysis.

**# Overview of the Signaling Questions**

**
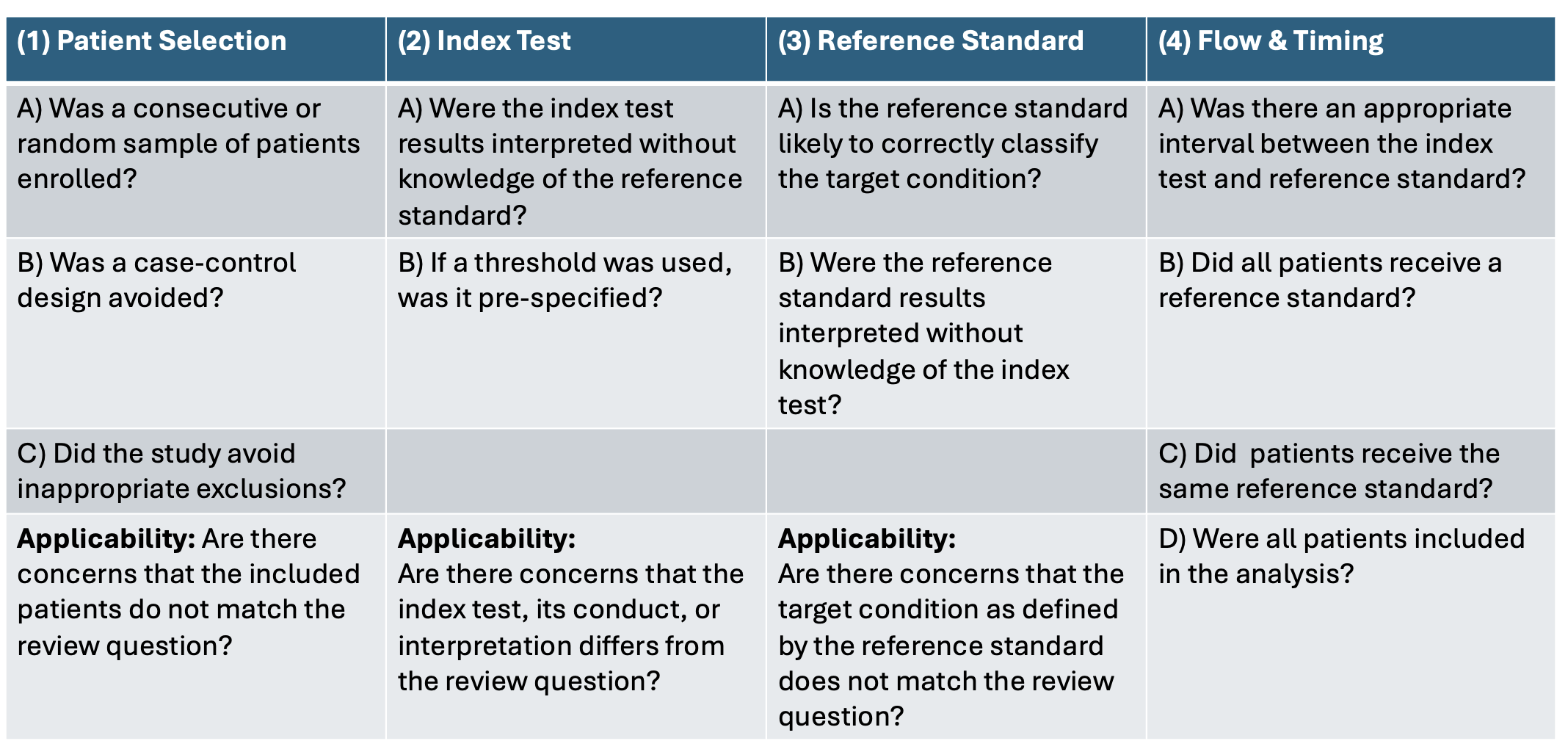
**

## **Patient selection**

### **1.1. Patient selection - Risk of Bias**

- Review and take notes on the definitions and inclusion criteria for ALS and control groups.
- Answer questions A–C as Yes/No/Unclear and determine the **overall Risk of Bias**. While **this is not intended to be a rigid scoring system**, the following criteria may serve as a general guide. **If there is a critical concern in any item, the overall risk rating may be upgraded accordingly.**
  - **LOW**: ≥ 2 Yes
  - **HIGH:** ≥ 2 No **OR** 1 No + 2 Unclear
  - **UNCLEAR:** 1 Yes + 1 No + 1 Unclear **OR** 1 Yes + 2 Unclear

**Signaling questions:**

1. **Was a consecutive or random sample of patients enrolled?**
   Answer: Yes/No/Unclear

- **Yes:** Explicitly mentioned that the sampling process was “consecutive” or “random.”
- **Unclear:** Not explicitly stated.

1. **Was a case-control design avoided?**Answer: Yes/No/Unclear

- **Yes:** The Study is an actual prospective diagnostic study
- **No:** Despite appearing to be a prospective cohort-like study design, it uses external controls or a retrospective design for diagnostic comparison.
- **Unclear:** Not applicable. It should generally be judged as either Yes or No.

*Note: Many biomarker studies employ a practical* ***case-control*** *design. An actual “prospective diagnostic study” begins with a cohort of suspected cases without knowledge of the final diagnosis and then compares index test results against the reference standard.* *Even if the study appears to be cohort-based, if the diagnostic value is assessed by comparing ALS patients to controls, it may be technically a case-control design.*

1. **Did the study avoid inappropriate exclusions?**Answer: Yes/No/Unclear
   - **Yes**: None of the conditions listed below apply.
   - **Unclear**: Applies in any of the **following situations**:
     - Exclusion criteria were not explicitly mentioned. If only the inclusion criteria are stated, and there is no statement indicating that all cases within specific time/facility/diagnostic criteria were included.
     - The eligible criteria for the control group are not clearly described.
     - The control group is defined as disease controls (e.g., AD, PD, myopathies, neuropathies), but their diagnostic criteria or recruitment process are not described.
   - **No**: Subjects were excluded based on biomarker results (e.g., outliers).

### **1.2. Patient selection - Concerns regarding applicability**

- Evaluate whether the inclusion of ALS patients reflects clinical practice. (HIGH/LOW/UNCLEAR)

**Applicability assessment question:**

**Are there concerns that the included patients do not match the review question?**Answer: HIGH/LOW/UNCLEAR

- **UNCLEAR**: ALS diagnostic criteria not clearly stated
- **LOW**: Clearly defined clinical criteria or citation provided ALS
  - For clinical diagnostic criteria, if it is not written in the text, it **should be cited**

## **2. Index Test**

### **2.1. Index test – Risk of Bias**

- Assess the methods and interpretation of the index test (biomarkers) for potential bias.
- Answer questions A–B as Yes/No/Unclear, then determine the overall Risk of Bias.
- While **this is not intended to be a rigid scoring system**, the following criteria may serve as a general guide. **If there is a critical concern in any item, the overall risk rating may be upgraded accordingly.**
  - **LOW**: ≥ 1 Yes
  - **HIGH**: any other combinations

**Signaling questions:**

1. **Were the index test results interpreted without knowledge of the reference standard?**
   Answer: Yes/No/Unclear

- **Yes**: If the condition below does not apply.
- **Unclear**: If it is not clearly stated that technicians or analysts were blinded to the reference standard.

1. **If a threshold was used, was it pre-specified?**Answer: Yes/No/Unclear

- **Yes**:
  - If the threshold was predefined based on prior studies.
  - If a test set and validation set were used within the same study, and the cutoff defined in the test set was applied to the validation set, with diagnostic performance reported.
- **No**: If thresholds were defined post hoc based on ROC analysis (e.g., maximizing Youden index, highest positive value), and not validated in an independent (external) cohort.
- **Unclear**: Should generally be judged as either Yes or No.

### **2.2. Index test – Concerns regarding applicability**

- Assess whether the index test, its conduct, or interpretation aligns with the review question. **(**HIGH/LOW/UNCLEAR**)**

**Applicability assessment question:**

**Are there concerns that the index test, its conduct, or interpretation differs from the review question?**

- **HIGH:** Considered high risk when reproducibility across different laboratories is difficult to confirm. Examples include the following:
  - Assays not commercially available
  - Studies that propose diagnostic models integrating multiple biomarkers—such as those derived from omics data using machine learning techniques—may pose concerns regarding reproducibility and applicability in other laboratories.
- **LOW**: If the above criteria are not met.

## **3. Reference Standard**

### **3.1. Reference standard - Risk of Bias**

- Assess how ALS was diagnosed.
- Determine the **overall Risk of Bias** based on responses to A and B.
- While this is **not intended to be a rigid scoring system**, the following criteria may serve as a general guide. If there is a critical concern in any item, **the overall risk rating may be upgraded accordingly**.
  - **LOW**: 2 Yes
  - **UNCLEAR**: 1 Yes + 1 Unclear **OR** 2 Unclear
  - **HIGH**: No ≥ 1

**Signaling questions:**

1. **Is the *reference standard* likely to correctly classify the target condition?**Answer: Yes/No/Unclear

- **Yes**: If the ***reference standard*** is based on established clinical criteria (explicitly stated or cited), genetic testing, or pathological confirmation.
- **Unclear**: If none of the above are satisfied (e.g., criteria not described or cited).
- **No**: If the ALS diagnosis is based solely on electronic medical records (EMR) or diagnostic codes, as is often the case in real-world data studies.

1. **Were the *reference standard* results interpreted without knowledge of the index test?**Answer: Yes/No/Unclear

- **Yes**: If the ***reference standard*** is based on established clinical diagnostic criteria for ALS (e.g., revised El-Escorial criteria, Awaji Criteria, and Gold Coast Criteria), which by definition do not incorporate biomarker values, it is reasonable to assume that the ***reference standard*** was interpreted without knowledge of the index test (=biomarker testing). The same applies when the ***reference standard*** is based on genetic testing or pathological confirmation.
- **Unclear**: If it is unclear whether a consistent reference standard was practically applied in the clinical setting (e.g., diagnoses based on electronic medical records without specification of the applied criteria, or reliance on ICD codes).

### **3.2. Reference standard – Concerns regarding Applicability:**

- Judge applicability based on how the ALS diagnosis was defined.

**Applicability assessment question:**

**Are there concerns that the target condition, as defined by the reference standard, does not match the review question?** Answer: HIGH/LOW/UNCLEAR

- **LOW**: If the diagnosis is based on the established clinical criteria (explicitly stated or cited), genetic testing, or pathological confirmation.
- **UNCLEAR**: If the clinical criteria are used but not clearly described or cited.
- **HIGH**: If no diagnostic criteria are specified.

## **4. Flow and Timing**

### **4.1. Flow and Timing - Risk of Bias:**

- Review the timing of biomarker testing, whether all patients received the reference standard, whether it was consistent across patients, and whether all were included in the analysis.
- Determine overall Risk of Bias based on responses to A–D.
- While this is **not intended to be a rigid scoring system**, the following criteria may serve as a general guide. If there is a critical concern in any item, **the overall risk rating may be upgraded accordingly**.
  - **LOW**: At least 3 Yes responses
  - **UNCLEAR**: 2 Yes + 2 Unclear **OR** 1 Yes + 3 Unclear, **OR** 4 Unclear
  - **HIGH**: Any other combinations

**Signaling questions:**

1. **Was there an appropriate interval between the index test and the reference standard?**Answer: Yes/No/Unclear

- **Yes**: If the testing occurred **within 3 months** from diagnosis or first evaluation
- **No**: If the interval was longer than 3 months or the study clearly included many late-stage patients
- **Unclear**: If it is not clearly stated when the samples were collected

1. **Did all patients receive a reference standard?**Answer: Yes/No/Unclear

- **Yes**: If the diagnosis of ALS (according to specific definitions) was determined in all patients.
  - *In practice, this should apply to most studies included in this systematic review.*
- **No**: If it is clear that not all patients received the reference standard.
- **Unclear**: It is unclear from the manuscript.

1. **Did all patients receive the same reference standard?**Answer: Yes/No/Unclear

- **Yes**: Consistent ALS criteria were used across the study.
- **No**: It is clear that not all patients received the same diagnostic criteria.
- **Unclear**: A multi-center cohort is used, and consistency across centers is not described.

1. **Were all patients included in the analysis?**Answer: Yes/No/Unclear

- **Yes:** It is clear that all patients were included in the diagnostic accuracy calculations (e.g., sensitivity, specificity, AUC).
- **No**: It is clear that not all data were used (e.g., mismatch between *N* in Table 1 and final sample sizes).
- **Unclear**: It is not clearly reported.
