# Supplementary material for: Diagnostic and Prognostic Value of Blood and Cerebrospinal Fluid Biomarkers in Amyotrophic Lateral Sclerosis: A Systematic Review and Meta‐Analysis
Source: Eur J Neurol. 2025 Oct 27;32(10):e70382. doi: 10.1111/ene.70382 (PMC12554952; doi:10.1111/ene.70382)
Supplement: Supplementary file 3 — Appendix S3: QUIPS tool Protocol for the ALS Biomarker Systematic Review and Meta‐Analysis. [file ENE-32-e70382-s006.docx]

This document outlines the prespecified protocol for applying the Quality in Prognosis Studies (QUIPS) tool. QUIPS assesses the risk of bias in studies evaluating prognostic factors, focusing on six specific bias domains. Each domain includes clearly defined prompting items to guide the assessment (Hayden et al., Ann Intern Med. 2013;158(4):280–286).

KO and DI developed the protocol for this systematic review. An initial version was drafted prior to the completion of data extraction and finalized after discussion. The finalized protocol was applied consistently to all prognostic studies included in the Cox hazard ratio meta-analysis.

# **Six domains in QUIPS**

| **Domains** | **Description** | **When High RoB*** |
| --- | --- | --- |
| 1. Study Participation | Assess whether the study sample adequately represents the population of interest | The relationship between the PF and outcome is very likely to be different for **participants** and **eligible nonparticipants** |
| 1. Study Attrition | Assess whether the study data available (i.e.,  participants not lost to follow-up) adequately represent the study sample | The relationship between the PF and outcome is very likely to be different for **completing** and **non-completing participants** |
| 1. Prognostic Factor Measurement | Assess whether the prognostic factor (PF) is measured in a similar way for all participants | The measurement of the PF is very likely to be different for different levels of the outcome of interest |
| 1. Outcome Measurement | Assess whether the outcome of interest measured in a similar way for all participants | The measurement of the outcome is very likely  to be different related to the baseline level of the PF |
| 1. Study Confounding | Assess whether the important potential confounding factors are appropriately accounted for | The observed effect of the PF on the outcome is very likely to be **distorted by another factor related to PF and outcome** |
| 1. Statistical Analysis and Reporting | Assess whether the statistical analysis is  appropriate, and all primary outcomes are reported | The reported results are very likely to be spurious or biased related to analysis or reporting |

* When Moderate RoB: very likely 🡪 may be
* When Low RoB: very likely 🡪 unlikely

## **#1. Study Participation**

| **Prompting Items** | **Judgment Criteria** | **Yes** | **No** | **Unclear** | **Critical** |
| --- | --- | --- | --- | --- | --- |
| **a. Adequate participation in the study by eligible persons** | Is the number of eligible vs. included ALS patients reported or estimable? (e.g., showing Patient Flow Diagram) | Clearly described or estimable | Not described | Vaguely described or partially reported | - |
| **b. Description of the source population or population of interest** | Are ALS diagnostic criteria explicitly stated or cited (e.g., El Escorial, Gold Coast)? | Clearly stated or cited | Implied only, not stated or cited | - | **YES** |
| **c. Description of the baseline study sample** | Is the number of ALS patients (sample size) included in survival analysis clearly reported? | Total number clearly reported | Not reported | Ambiguous or inconsistently reported (e.g., reported only in univariate model but not in multivariate model) | - |
| **d. Adequate description of the sampling frame and recruitment** | Is the recruitment method (e.g., random, consecutive, convenience) clearly described? | Clearly described | Not described | Vague or implied | - |
| **e. Adequate description of the period and place of recruitment** | Are both the recruitment dates and clinical setting reported? | Both reported (e.g., “2012–2018 at X Hospital”) | Neither reported | Only one reported or vaguely described | - |
| **f. Adequate description of inclusion and exclusion criteria** | Are criteria clearly defined, and can exclusions of eligible ALS cases (e.g., due to missing CSF/blood) be identified? | Clearly defined with rationale for exclusions | Criteria or exclusions not described | Vaguely described or partially reported | - |

- While **this is not intended to be a rigid scoring system**, the following criteria may serve as a general guide.
  - Low Risk: 0–2 prompting items are rated as “No” or “Unclear”.
  - Moderate Risk: 3–4 prompting items are rated as “No” or “Unclear”.
  - High Risk: 5–6 prompting items are rated as “No” or “Unclear”.
  - If **critical item (b)** is "No" or "Unclear", rate the overall risk one level higher (i.e., low -> moderate, moderate -> high).

## **#2. Study Attrition**

- Note) In a retrospective study, most items may inherently be “no” or “unclear.”

| **Prompting Items** | **Judgment Criteria** | **Yes** | **No** | **Unclear** | **Critical** |
| --- | --- | --- | --- | --- | --- |
| **a. Adequate response rate for study participants** | Is the proportion of patients with complete survival follow-up reported and sufficient (e.g., outcome date available for most)? | Proportion reported and clearly high (e.g., ≥80% follow-up to death or censoring because of the study period) | Follow-up proportion low or evidently incomplete | Not reported or cannot be calculated | - |
| **b. Description of attempts to collect information on participants who dropped out** | Are attempts described to trace participants lost to follow-up (e.g., phone contact, national registries)? | Active follow-up or data linkage described | No attempt to follow-up missing participants described | Not mentioned | - |
| **c. Reasons for loss to follow-up are provided** | Are reasons for dropout or incomplete follow-up described (e.g., withdrawal, relocation, inability to contact)? | Reasons stated for all or most lost cases | Losses reported but no reasons provided | Not mentioned about lost to follow-up | - |
| **d. Adequate description of participants lost to follow-up** | Are characteristics of patients lost to follow-up compared with those retained (e.g., age, ALSFRS-R)? | Baseline comparison presented | Not compared | Not mentioned or unclear | - |
| **e. There are no important differences between participants who completed the study and those who did not** | Are differences assessed and found to be minimal or unlikely to bias results? **If (d) is “unclear” or “no”, this item will be automatically “no”.** | Stated explicitly or can be inferred from data | Clear differences suggesting attrition bias | Not assessed or unclear | - |

- While **this is not intended to be a rigid scoring system**, the following criteria may serve as a general guide.
  - Low Risk: 0–1 prompting items are rated as “No” or “Unclear”.
  - Moderate Risk: 2–3 prompting items are rated as “No” or “Unclear”.
  - High Risk: 4–5 prompting items are rated as “No” or “Unclear”.

## **#3: Prognostic Factor Measurement**

| **Prompting Items** | **Judgment Criteria** | **Yes** | **No** | **Unclear** | **Critical** |
| --- | --- | --- | --- | --- | --- |
| **a. A clear definition or description of the PF is provided** | Is the biomarker clearly defined? (e.g., baseline plasma NfL, CSF TDP-43) | Biomarker fully defined | Somewhat unclear definition | - | **-** |
| **b. Method of PF measurement is adequately valid and reliable** | Were the measurement technique and assays explicitly described (e.g., ELISA, Simoa, MSD…)? | Valid method described | Method not clearly validated | Not specified | - |
| **c. Continuous variables are reported, or appropriate cut points are used** | Is the biomarker treated as a continuous variable, or categorized using **predefined** thresholds (not based on study-specific data) **in statistical models? (e.g., Cox model)** | Reported continuously or predefined cutoffs (e.g., clinical thresholds) | Cut points chosen **post hoc** or **data-driven** (e.g., median split, tertile split) | Not reported or unclear | - |
| **d. The method and setting of measurement of PF is the same for all study participants** | Were all samples analyzed using the same protocol (e.g., same assay, same storage conditions, same platform)? | Uniform method described in Methods section | Different methods used without validation/rationale | Not specified | - |
| **e. Adequate proportion of the study sample has complete data for the PF** | Was biomarker data available for nearly all participants included in the survival analysis? | Complete or near-complete data (>90%) | Large proportion of cases excluded due to missing PF | Not reported | - |
| **f. Appropriate methods of imputation are used for missing PF data** | If imputation was done, was it reported and appropriate (e.g., multiple imputation)? | Appropriate method used and clearly reported | not reported or unjustified imputation used | No imputation applied | - |

- While **this is not intended to be a rigid scoring system**, the following criteria may serve as a general guide.
  - Low Risk: 0–2 prompting items are rated as “No” or “Unclear”.
  - Moderate Risk: 3–4 prompting items are rated as “No” or “Unclear”.
  - High Risk: 5–6 prompting items are rated as “No” or “Unclear”.

## **#4: Outcome Measurement**

| **Prompting Items** | **Judgment Criteria** | **Yes** | **No** | **Unclear** | **Critical** |
| --- | --- | --- | --- | --- | --- |
| **a. A clear definition of the outcome is provided** | Is the outcome explicitly stated (e.g., “time to death,” “time to death or tracheostomy”)? | Clearly defined in Methods or Outcome section | Not defined | Implicit or inconsistent terminology | - |
| **b. Method of outcome measurement used is adequately valid and reliable** | Since all included studies set the endpoint as death or death/IMV, **this item will be uniformly satisfied across studies** | Death/IMV used as endpoint | Subjective or unclear definition (e.g., "poor outcome") | Unclear how outcome was recorded | - |
| **c. The method and setting of outcome measurement is the same for all study participants** | Was the same source used to ascertain outcomes for all (e.g., EMR, registry, structured follow-up)? | Consistent across all patients | Obviously different methods applied (e.g., registry for some, phone calls for others) | Unclear | - |
| **d. The outcome is measured in a similar way for all participants** | Was the outcome applied uniformly (e.g., same censoring rules, same endpoint definition)? | Uniform definitions and censoring rules across the cohort | Differential censoring, or outcome definitions vary | Not reported | - |
| **e. Adequate follow-up duration to observe the outcome** | Is the median follow-up period long enough to reasonably observe the outcome in most patients? | Median Follow-up ≥1 years | Median Follow-up <1 year without justification | Duration not reported or unclear | **YES** |

- While **this is not intended to be a rigid scoring system**, the following criteria may serve as a general guide.
- Low Risk: 0–2 prompting items are rated as “No” or “Unclear”.
- Moderate Risk: 3–4 prompting items are rated as “No” or “Unclear”.
- High Risk: 5 prompting items are rated as “No” or “Unclear”.
- If **critical item (e)** is "No" or "Unclear", rate the overall risk one level higher (i.e., low -> moderate, moderate -> high).

## **#5: Study Confounding**

| **Prompting Items** | **Judgment Criteria** | **Yes** | **No** | **Unclear** | **Critical** |
| --- | --- | --- | --- | --- | --- |
| **a. All important confounders are measured** | Were **age** and at least **two** key **ALS-specific prognostic factor** (listed below) included in the data collection? (likely in Table 1) | Age + ≥ 2 known factors (below) measured | Age missing or < 2 known prognostic factors measured | Not reported or insufficient detail | **YES** |
| **b. Clear definitions of the important confounders measured are provided** | Were variables clearly defined (e.g., baseline ALSFRS-R at diagnosis; age at sampling; disease duration from onset)? | Operational definitions provided | Definitions missing or ambiguous | Vaguely stated | - |
| c. Measurement of all important confounders is adequately valid and reliable | Excluded, as core clinical predictors are standardized variables (e.g., age, DPR, %FVC), making assessment of measurement validity largely redundant. | ~~-~~ | ~~-~~ | ~~-~~ | ~~-~~ |
| **d. The method and setting of confounding measurement are the same for all study participants** | Were confounders measured in the same way across all participants (e.g., biomarker measurement and baseline assessment timing are not different across patients)? | Measurement consistent across cohort (e.g., clear timeframe from sampling to baseline assessment) | Known inconsistency (e.g., ALSFRS-R measured at different disease phases) | Not stated | - |
| **e. Appropriate methods are used if imputation is used for missing confounder data** | If imputation was applied to missing confounder data, were methods appropriate (e.g., multiple imputation, sensitivity analyses)? | Missing data handled transparently and appropriately | Missing confounders ignored or inappropriately imputed | Missing data mentioned but method unclear | - |
| f. Important potential confounders are accounted for in the study design | Not applicable in this review, since the included studies are mostly retrospective or cross-sectional research. | ~~-~~ | ~~-~~ | ~~-~~ | ~~-~~ |
| **g. Important potential confounders are accounted for in the analysis** | Were **age** and at least **two prognostic factors included in multivariable models**, or was subgroup/sensitivity analysis accounting for them performed? | Adjusted for age and relevant ≥ 2 confounders | Not adjusted for age and relevant ≥ 2 confounders | Model unclear or poorly reported | - |

- While **this is not intended to be a rigid scoring system**, the following criteria may serve as a general guide.
  - Low Risk: 0–2 prompting items are rated as “No” or “Unclear”.
  - Moderate Risk: 3–4 prompting items are rated as “No” or “Unclear”.
  - High Risk: 5 prompting items are rated as “No” or “Unclear”.

Key ALS-specific prognostic confounder:
**Age + at least ≥ 2 factors (listed below) should be measured and included in the multivariate models**

**Core clinical predicters** (cf. ENCALS linear predictive model score; Westeneng HJ, et al. Lancet Neurol. 2018;17(5):423-433.)

1. Age
2. disease duration or diagnostic delay
3. site of onset (e.g., bulbar onset vs spinal onset)
4. DPR (∆FS), or baseline ALSFRS-R
5. diagnostic classification (e.g., El-Escorial definite)
6. %FVC (or %SVC)
7. FTD status (presence of FTD)
8. Presence of C9orf72 repeat expansion

- + Other mutation profile

## **#6: Statistical Analysis and Reporting**

| **Prompting Items** | **Judgment Criteria** | **Yes** | **No** | **Unclear** | **Critical** |
| --- | --- | --- | --- | --- | --- |
| **a. Sufficient presentation of data to assess the adequacy of the analytic strategy** | Are essential details of the analysis provided (e.g., number of events, total sample size, HRs with 95% CI, **all covariates** in the model)? | Model, **covariates**, and summary stats (e.g., HR, CI) clearly described | HRs or models reported with no indication of model structure or events | Model used, but structure or data insufficiently reported | - |
| **b. Strategy for model building is appropriate and is based on a conceptual framework or model** | Does the study explain **how covariates were selected** (e.g., prior knowledge, clinical reasoning, univariate screening, data-driven)? | Justified variable selection strategy; **based on prior knowledge (citing previous studies) or prespecified protocol** | Arbitrary or data-driven approach with no validation/ justification (e.g., stepwise) | Not discussed | - |
| **c. The selected statistical model is adequate for the design of the study** | Was an appropriate time-to-event method (e.g., multivariable Cox model) used? | Cox proportional hazards model. | Unjustified models | Not specified | - |
| **d. There is no selective reporting of results** | Are all models described in Methods also reported in Results? Were **sensitivity analyses performed*** (e.g., different covariate sets, biomarker cutoffs)? | Sensitivity or subgroup analyses shown or discussed; All stated analyses reported | Some models described but not reported; only single model reported without rationale | Unclear whether additional analyses were conducted or omitted. (e.g., **only one model is reported**) | - |

*) Ideally, multivariable models with different covariate combinations (sensitivity analyses) should be reported.

- While **this is not intended to be a rigid scoring system**, the following criteria may serve as a general guide.
  - Low Risk: 0–1 prompting items are rated as “No” or “Unclear”.
  - Moderate Risk: 2–3 prompting items are rated as “No” or “Unclear”.
  - High Risk: 4 prompting items are rated as “No” or “Unclear”.
