# Supplementary figures and images for: Diagnostic and Prognostic Value of Blood and Cerebrospinal Fluid Biomarkers in Amyotrophic Lateral Sclerosis: A Systematic Review and Meta‐Analysis
Source: Eur J Neurol. 2025 Oct 27;32(10):e70382. doi: 10.1111/ene.70382 (PMC12554952; doi:10.1111/ene.70382)

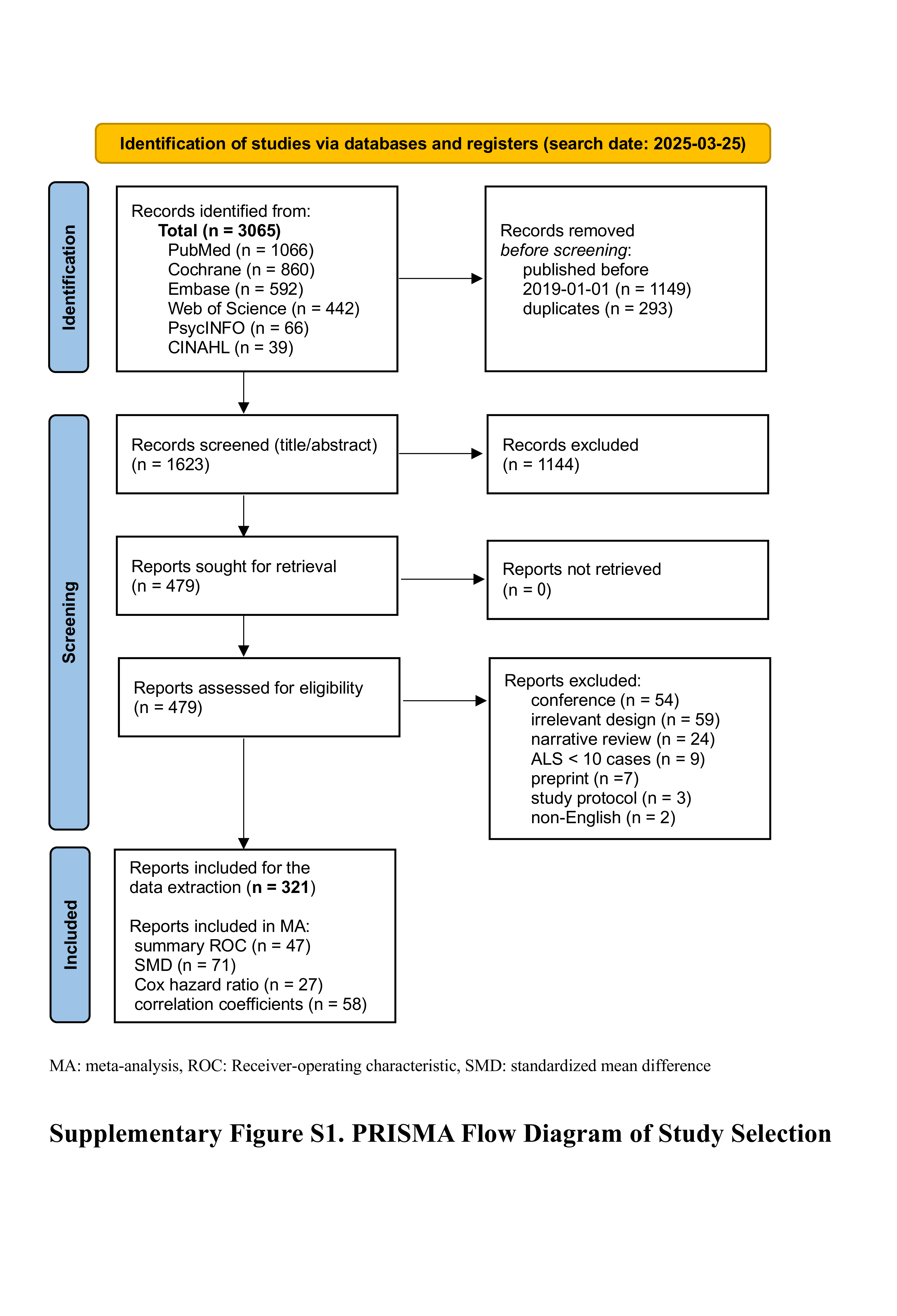

Supplement: Supplementary file 5 — Figure S1: PRISMA Flow Diagram of Study Selection. The diagram outlines the number of records identified, screened, assessed for eligibility, and included in the final analysis, following the PRISMA 2020 guidelines. [file ENE-32-e70382-s011.png]

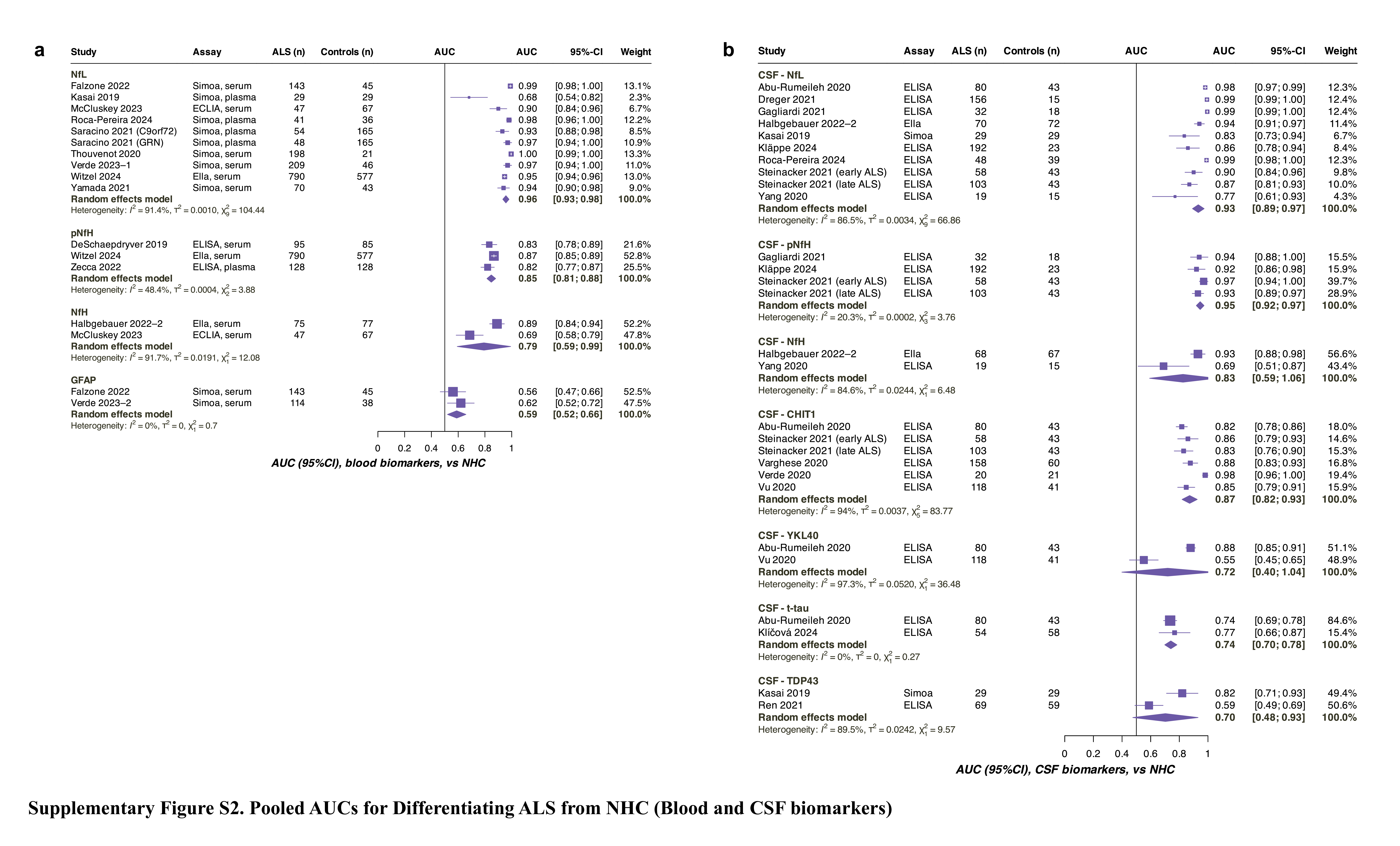

Supplement: Supplementary file 6 — Figure S2: Pooled AUCs for Differentiating ALS from NHC (Blood and CSF Biomarkers). Panel (a) shows pooled areas under the receiver operating characteristic curves (AUCs) for blood biomarkers, and panel (b) shows those for CSF biomarkers, both estimated using random‐effects models. [file ENE-32-e70382-s012.png]

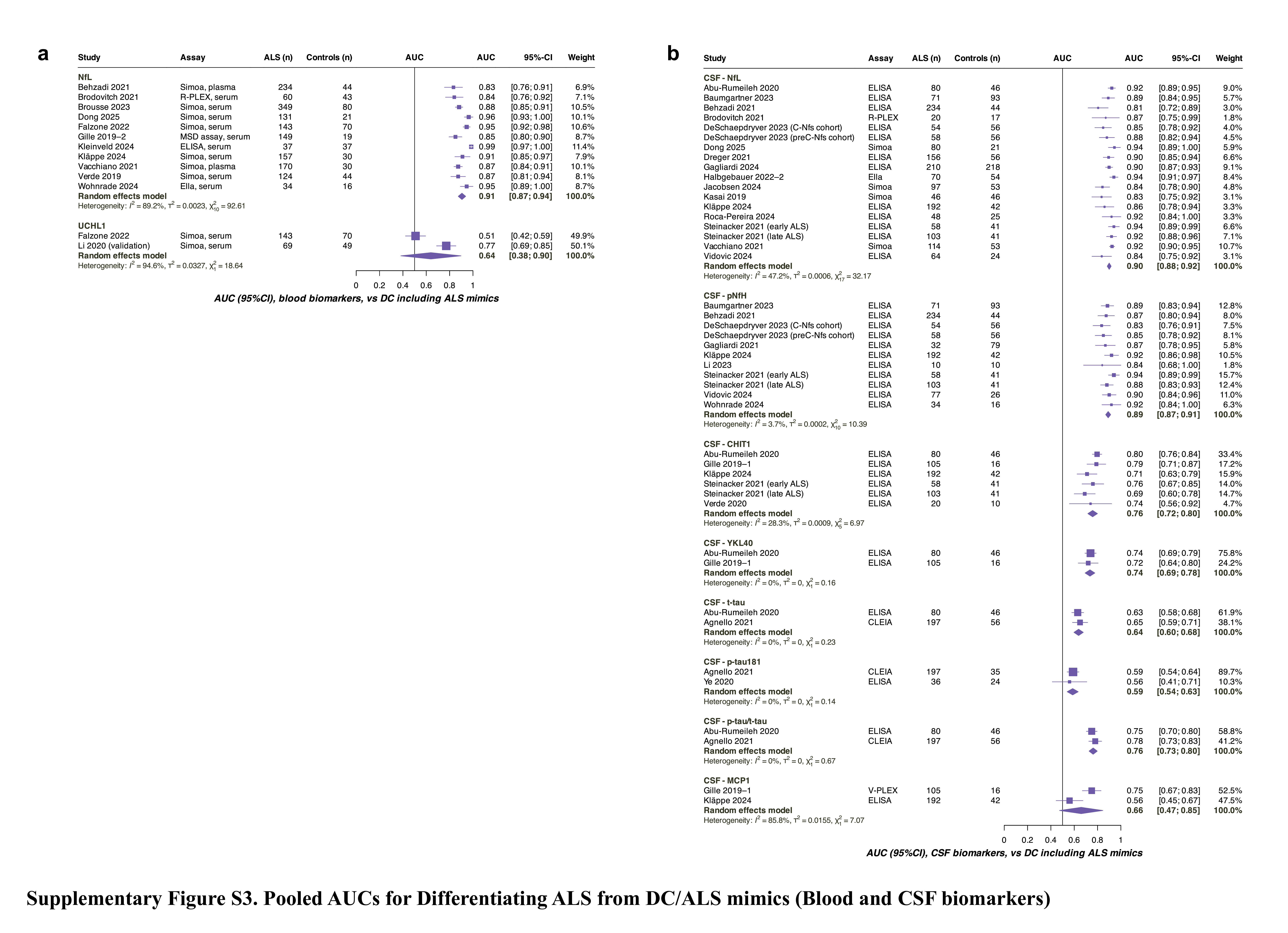

Supplement: Supplementary file 7 — Figure S3: Pooled AUCs for Differentiating ALS from DC/ALS Mimics (Blood and CSF Biomarkers). Panel (a) shows pooled areas under the receiver operating characteristic curves (AUCs) for blood biomarkers, and panel (b) shows those for CSF biomarkers, both estimated using random‐effects models. [file ENE-32-e70382-s003.png]

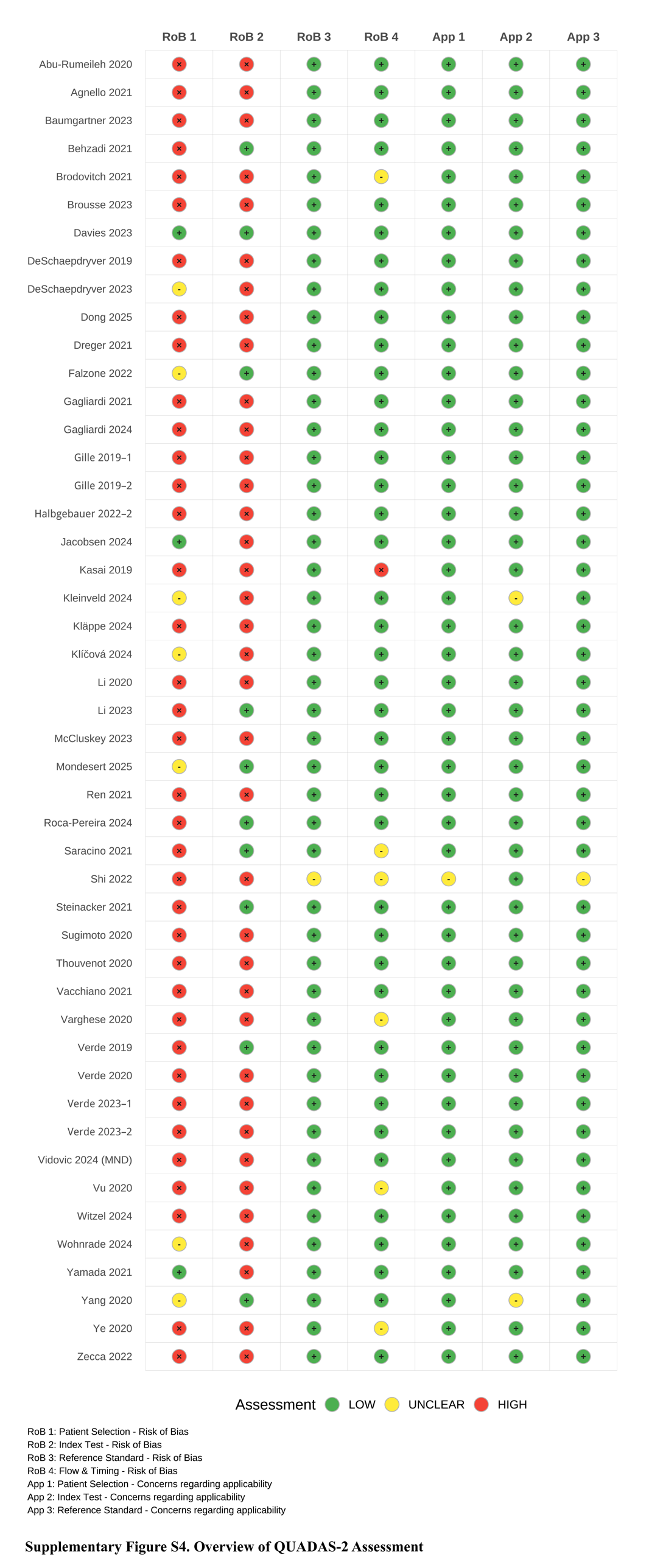

Supplement: Supplementary file 8 — Figure S4: Overview of QUADAS‐2 Assessment. The figure summarizes domain‐level judgments based on consensus between two independent reviewers, according to the QUADAS‐2 framework. QUADAS‐2, Quality Assessment of Diagnostic Accuracy Studies 2. [file ENE-32-e70382-s007.png]

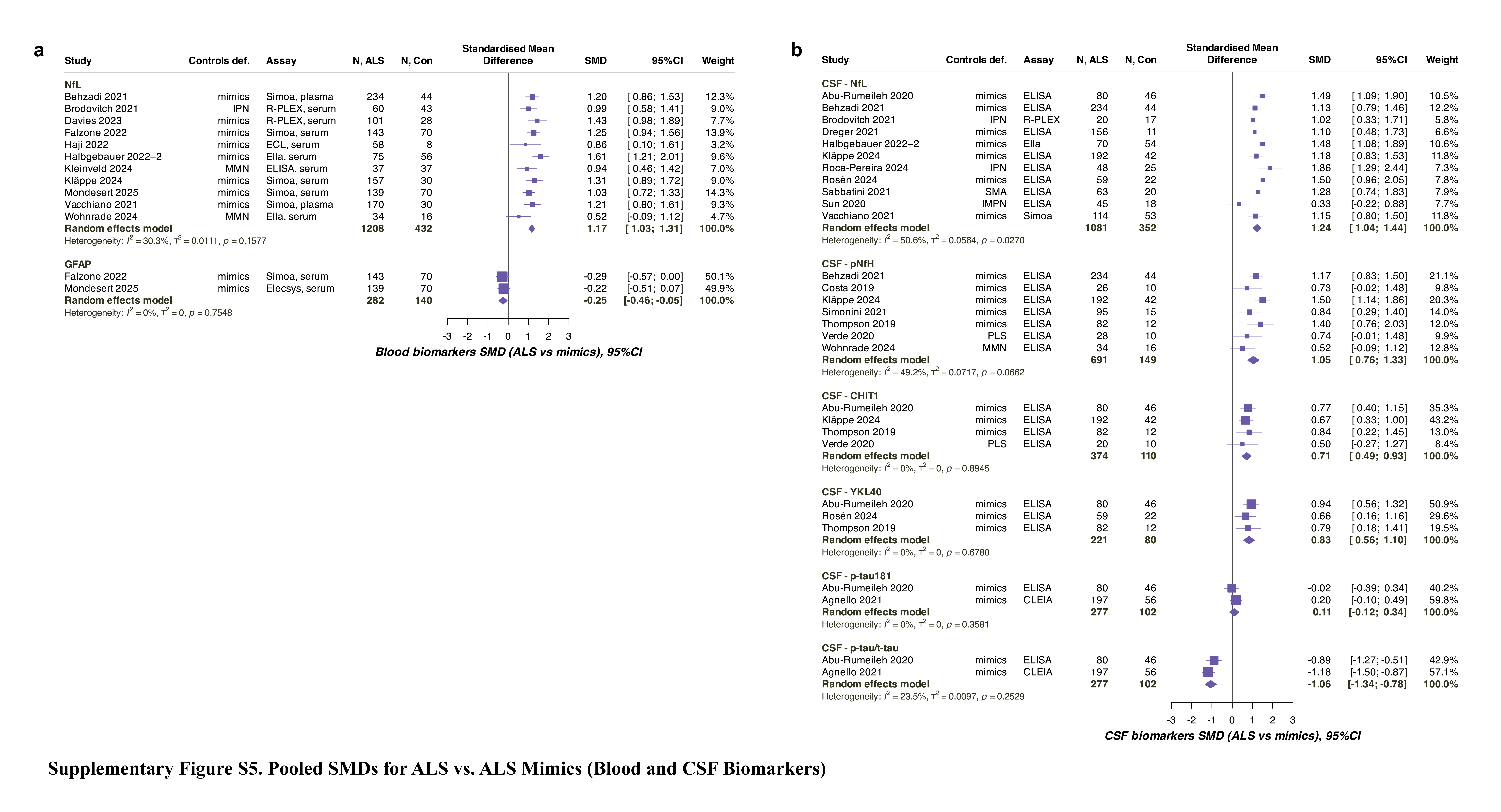

Supplement: Supplementary file 9 — Figure S5: Pooled SMDs for ALS vs. ALS Mimics (Blood and CSF Biomarkers). Panel (a) shows pooled Standardized Mean Differences (SMDs) for blood biomarkers, and panel (b) shows those for CSF biomarkers, both estimated using random‐effects models. [file ENE-32-e70382-s014.png]

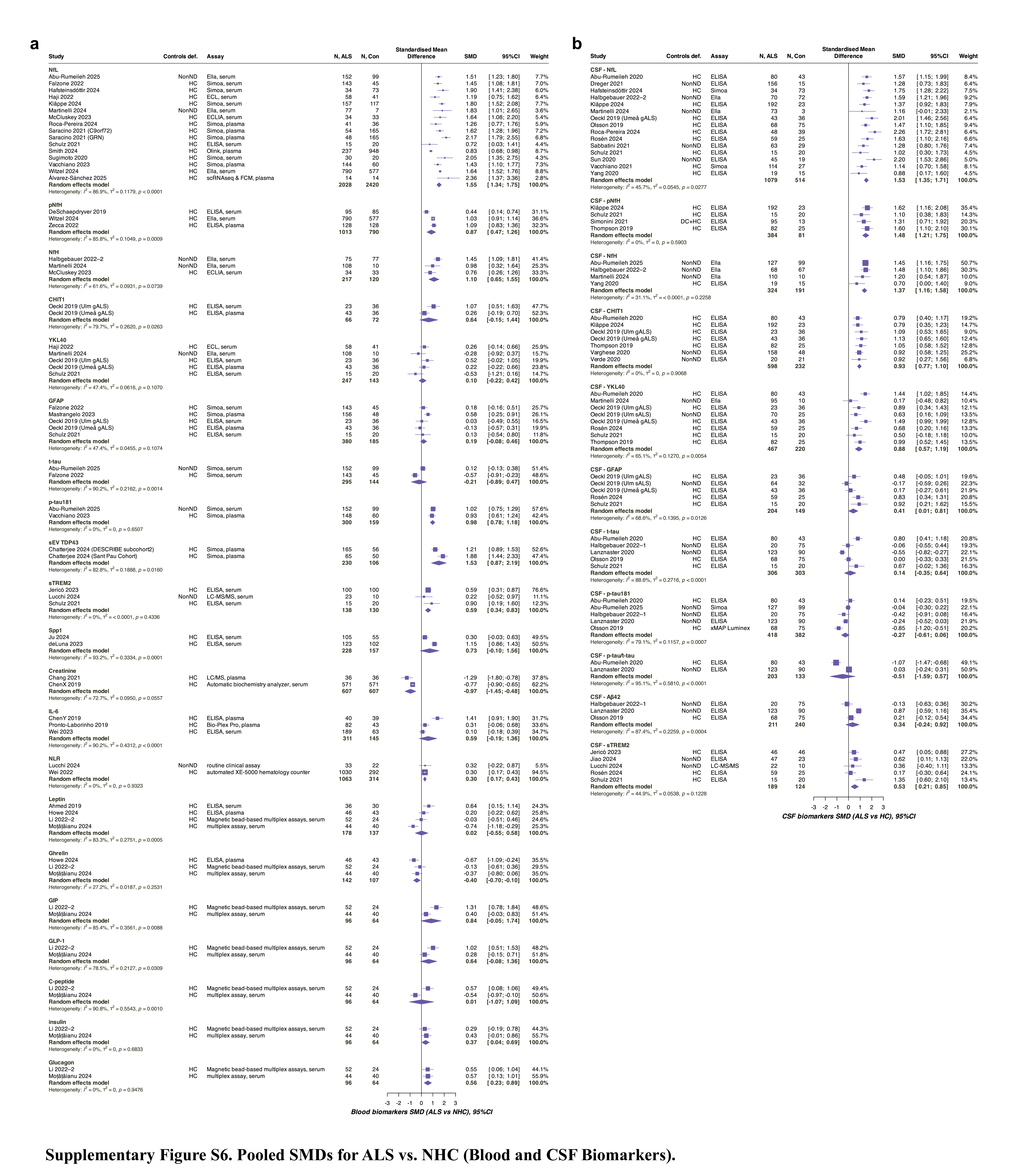

Supplement: Supplementary file 10 — Figure S6: Pooled SMDs for ALS vs. NHC (Blood and CSF Biomarkers). Panel (a) shows pooled Standardized Mean Differences (SMDs) for blood biomarkers, and panel (b) shows those for CSF biomarkers, both estimated using random‐effects models. [file ENE-32-e70382-s005.png]

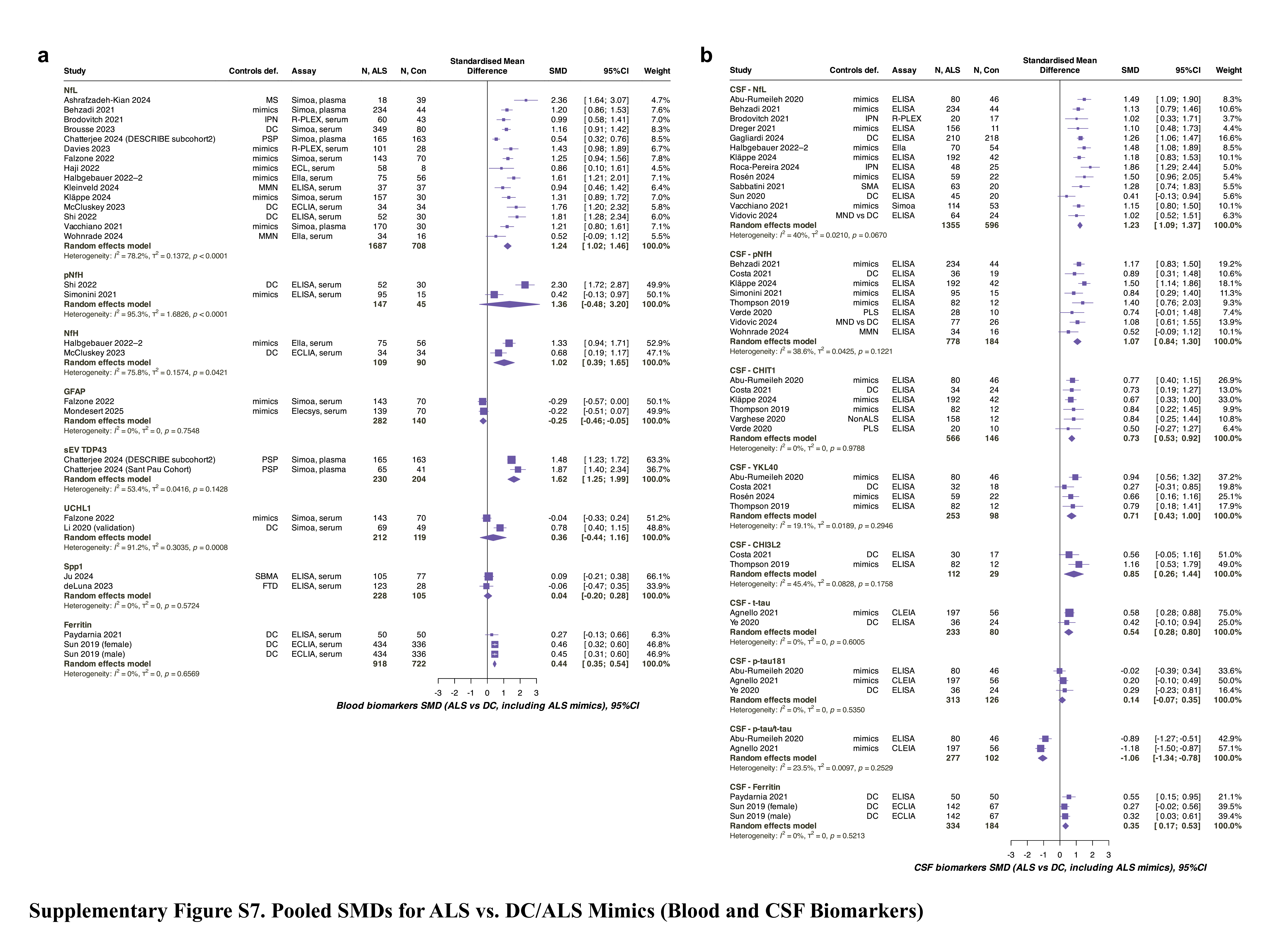

Supplement: Supplementary file 11 — Figure S7: Pooled SMDs for ALS vs. DC/ALS Mimics (Blood and CSF Biomarkers). Panel (a) shows pooled Standardized Mean Differences (SMDs) for blood biomarkers, and panel (b) shows those for CSF biomarkers, both estimated using random‐effects models. [file ENE-32-e70382-s016.png]

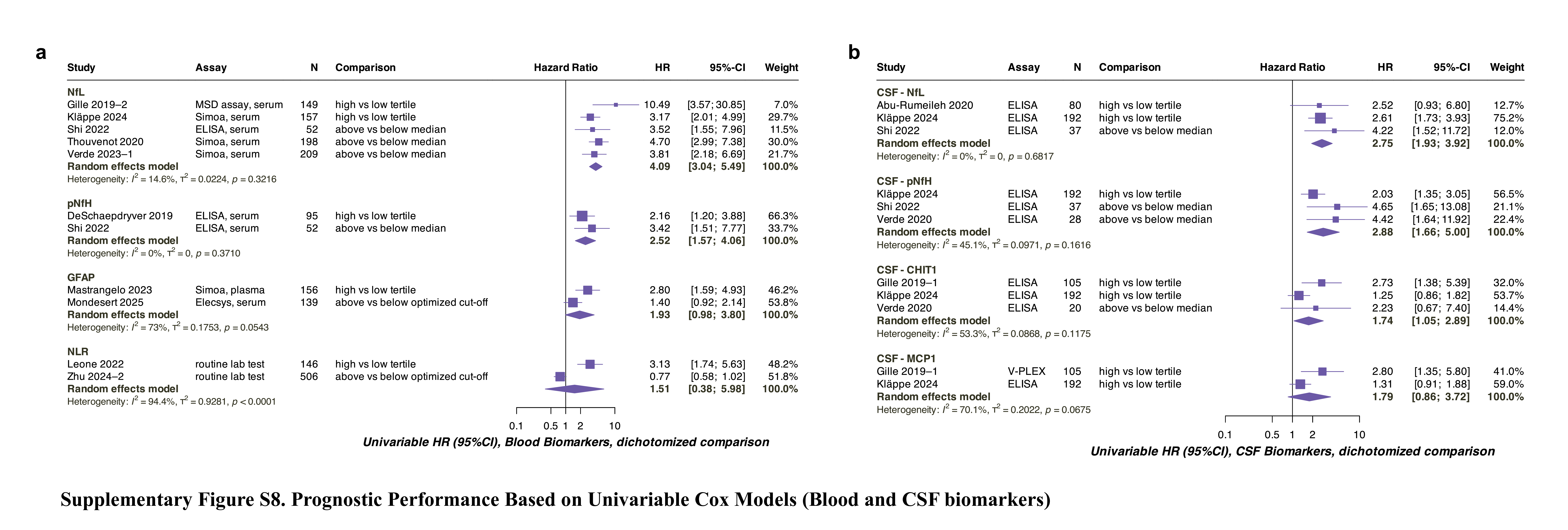

Supplement: Supplementary file 12 — Figure S8: Forest Plots of Pooled Univariable Cox Hazard Ratios (Blood and CSF Biomarkers). Panel (a) shows pooled hazard ratios (HRs) for blood biomarkers, and panel (b) shows those for CSF biomarkers, both estimated using random‐effects models. All HRs are derived from Cox proportional hazards models evaluating time to death or tracheostomy. [file ENE-32-e70382-s001.png]

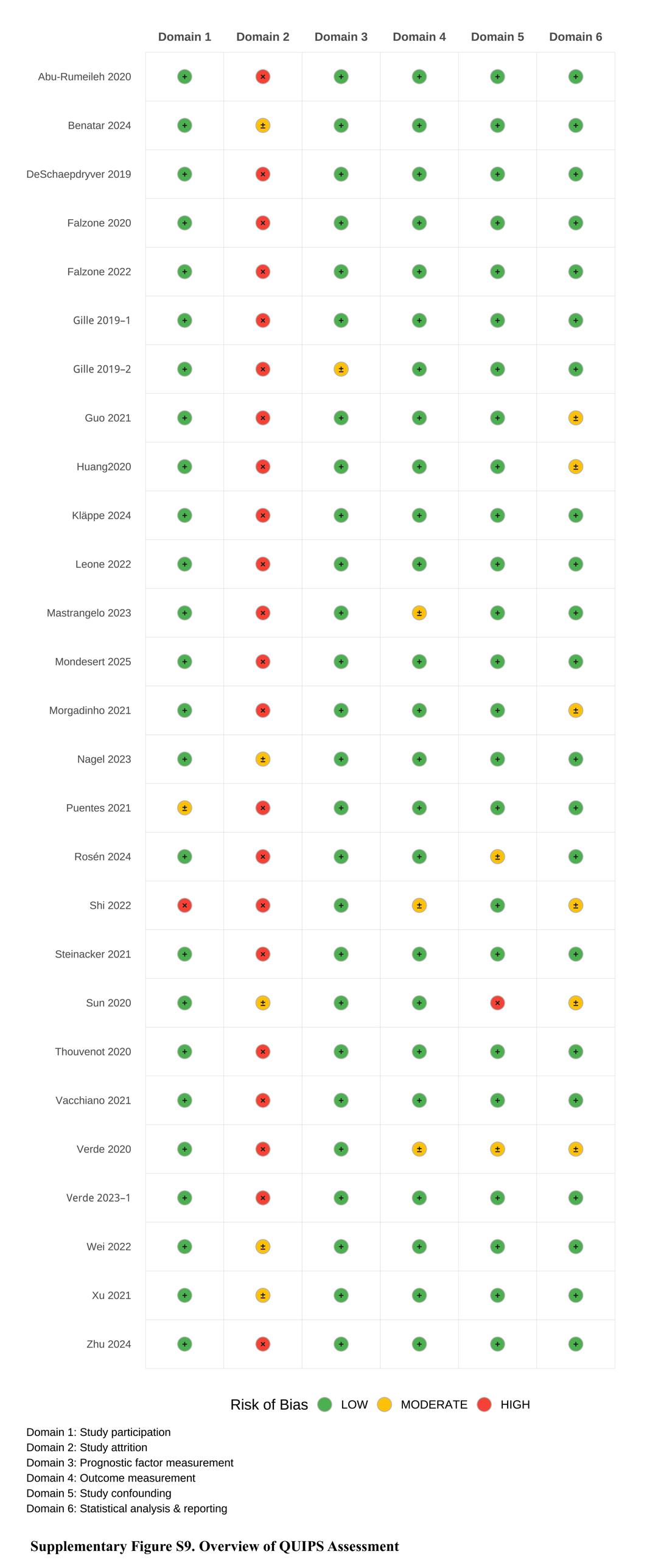

Supplement: Supplementary file 13 — Figure S9: Overview of QUIPS assessment. The figure summarizes domain‐level judgments based on consensus between two independent reviewers, according to the QUIPS framework. QUIPS, quality in prognosis studies. [file ENE-32-e70382-s008.png]

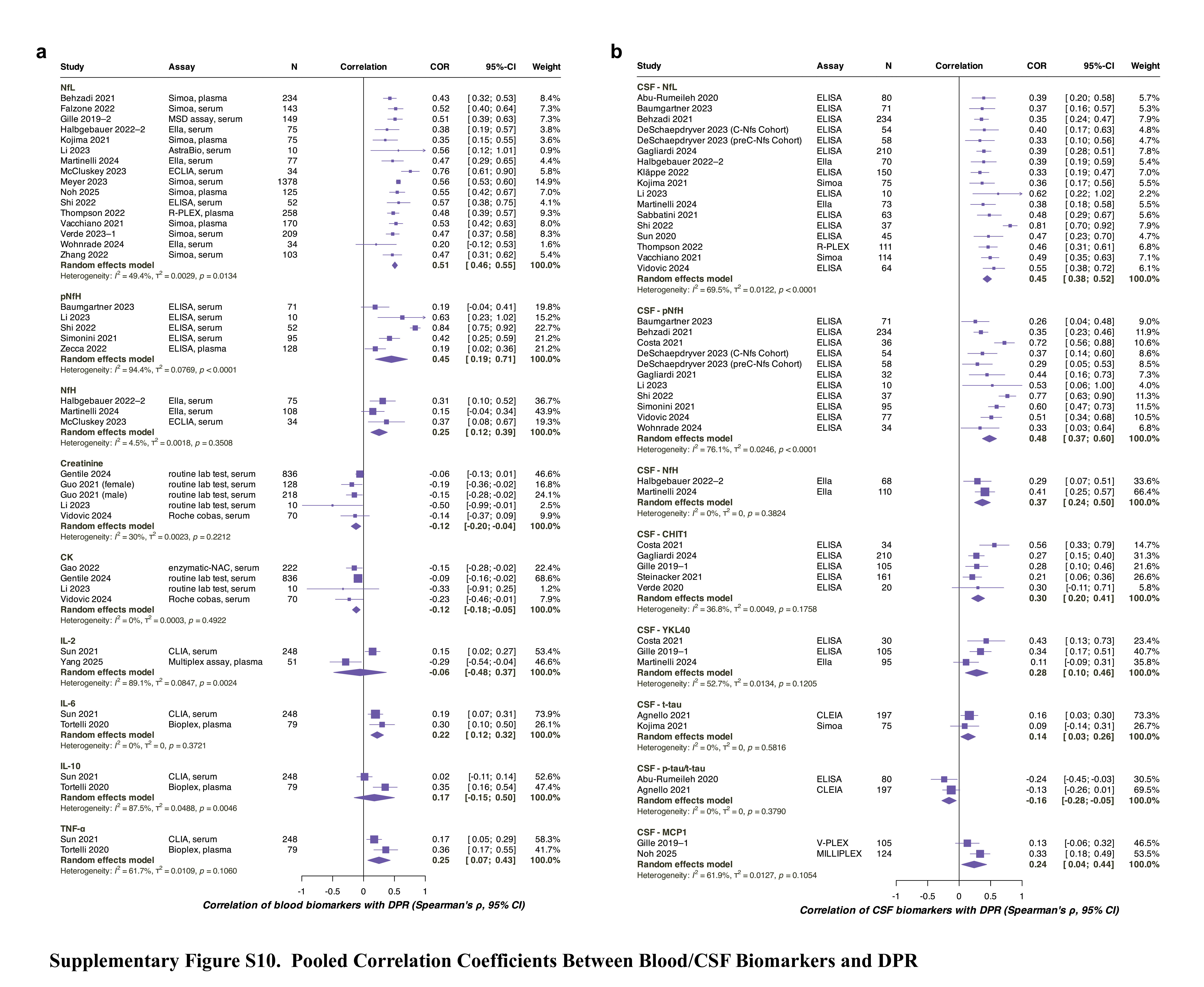

Supplement: Supplementary file 14 — Figure S10: Pooled Correlation Coefficients Between Blood/CSF Biomarkers and DPR. Panel (a) shows pooled correlation coefficients for blood biomarkers, and panel (b) shows those for CSF biomarkers, both estimated using random‐effects models. [file ENE-32-e70382-s002.png]

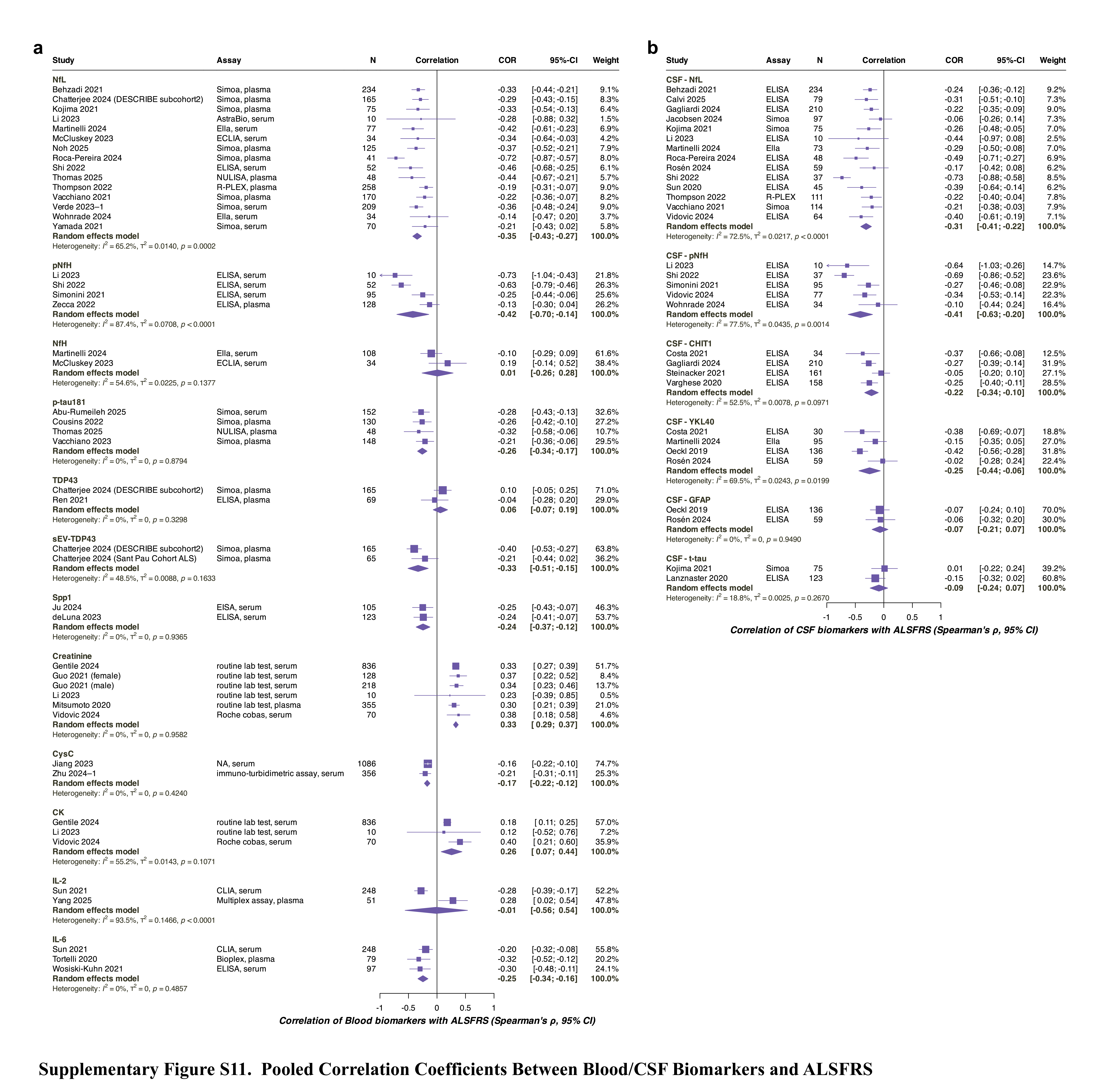

Supplement: Supplementary file 15 — Figure S11: Pooled Correlation Coefficients Between Blood/CSF Biomarkers and ALSFRS. Panel (a) shows pooled correlation coefficients for blood biomarkers, and panel (b) shows those for CSF biomarkers, both estimated using random‐effects models. [file ENE-32-e70382-s009.png]
