# Supplementary material for: Diagnostic and Prognostic Value of Blood and Cerebrospinal Fluid Biomarkers in Amyotrophic Lateral Sclerosis: A Systematic Review and Meta‐Analysis
Source: Eur J Neurol. 2025 Oct 27;32(10):e70382. doi: 10.1111/ene.70382 (PMC12554952; doi:10.1111/ene.70382)
Supplement: Supplementary file 16 — Table S1: Summary of pooled standardized mean differences (SMDs). Table S2: Covariates included in multivariable Cox regression models. [file ENE-32-e70382-s015.docx]

###### Supplementary Table S1. Summary of pooled standardized mean differences (SMDs)

|  | ***vs. Neurologically Healthy Controls*** | | | | ***vs. ALS mimics*** | | | | ***vs. Neurological Disease Controls (incl. mimics)*** | | | |
| --- | --- | --- | --- | --- | --- | --- | --- | --- | --- | --- | --- | --- |
| **Biomarker** | **Cohorts** | **N, ALS** | **N, Con** | **SMD [95%CI]** | **Cohorts** | **N, ALS** | **N, Con** | **SMD [95%CI]** | **Cohorts** | **N, ALS** | **N, Con** | **SMD [95%CI]** |
| **blood** |  |  |  |  |  |  |  |  |  |  |  |  |
| NfL | 16 | 2028 | 2420 | 1.55 [1.34; 1.75] | 11 | 1208 | 432 | 1.17 [1.03; 1.31] | 15 | 1687 | 708 | 1.24 [1.02; 1.46] |
| pNfH | 3 | 1013 | 790 | 0.87 [0.47; 1.26] | - | - | - | - | 2 | 147 | 45 | 1.36 [-0.48; 3.20] |
| NfH | 3 | 217 | 120 | 1.10 [0.65; 1.55] | - | - | - | - | 2 | 109 | 90 | 1.02 [0.39; 1.65] |
| CHIT1 | 2 | 66 | 72 | 0.64 [-0.15; 1.44] | - | - | - | - | - | - | - | - |
| YKL40 | 5 | 247 | 143 | 0.10 [-0.22; 0.42] | - | - | - | - | - | - | - | - |
| GFAP | 5 | 380 | 185 | 0.19 [-0.08; 0.46] | 2 | 282 | 140 | -0.25 [-0.46; -0.05] | 2 | 282 | 140 | -0.25 [-0.46; -0.05] |
| t-tau | 2 | 295 | 144 | -0.21 [-0.89; 0.47] | - | - | - | - | - | - | - | - |
| p-tau181 | 2 | 300 | 159 | 0.98 [0.78; 1.18] | - | - | - | - | - | - | - | - |
| sEV-TDP-43 | 2 | 230 | 106 | 1.53 [0.87; 2.19] | - | - | - | - | 2 | 230 | 204 | 1.62 [1.25; 1.99] |
| sTREM2 | 3 | 138 | 130 | 0.59 [0.34; 0.83] | - | - | - | - | - | - | - | - |
| Spp1 | 2 | 228 | 157 | 0.73 [-0.10; 1.56] | - | - | - | - | 2 | 228 | 105 | 0.04 [-0.20; 0.28] |
| UCHL1 | - | - | - | - | - | - | - | - | 2 | 212 | 119 | 0.36 [-0.44; 1.16] |
| IL-6 | 3 | 311 | 145 | 0.59 [-0.19; 1.36] | - | - | - | - | - | - | - | - |
| NLR | 2 | 1063 | 314 | 0.30 [0.17; 0.43] | - | - | - | - | - | - | - | - |
| Creatinine | 2 | 607 | 607 | -0.97 [-1.45; -0.48] | - | - | - | - | - | - | - | - |
| Leptin | 4 | 178 | 137 | 0.02 [-0.55; 0.58] | - | - | - | - | - | - | - | - |
| Ghrelin | 3 | 142 | 107 | -0.40 [-0.70; -0.10] | - | - | - | - | - | - | - | - |
| GIP | 2 | 96 | 64 | 0.84 [-0.05; 1.74] | - | - | - | - | - | - | - | - |
| GLP-1 | 2 | 96 | 64 | 0.64 [-0.08; 1.36] | - | - | - | - | - | - | - | - |
| C-peptide | 2 | 96 | 64 | 0.01 [-1.07; 1.09] | - | - | - | - | - | - | - | - |
| insulin | 2 | 96 | 64 | 0.37 [0.04; 0.69] | - | - | - | - | - | - | - | - |
| Glucagon | 2 | 96 | 64 | 0.56 [0.23; 0.89] | - | - | - | - | - | - | - | - |
| Ferritin | - | - | - | - | - | - | - | - | 3 | 918 | 722 | 0.44 [0.35; 0.54] |
| **CSF** |  |  |  |  |  |  |  |  |  |  |  |  |
| NfL | 15 | 1079 | 514 | 1.53 [1.35; 1.71] | 11 | 1081 | 352 | 1.24 [1.04; 1.44] | 13 | 1355 | 596 | 1.23 [1.09; 1.37] |
| pNfH | 4 | 384 | 81 | 1.48 [1.21; 1.75] | 7 | 691 | 149 | 1.05 [0.76; 1.33] | 8 | 778 | 184 | 1.07 [0.84; 1.30] |
| NfH | 4 | 324 | 191 | 1.37 [1.16; 1.58] | - | - | - | - | - | - | - | - |
| CHIT1 | 7 | 598 | 232 | 0.93 [0.77; 1.10] | 4 | 374 | 110 | 0.71 [0.49; 0.93] | 6 | 566 | 146 | 0.73 [0.53; 0.92] |
| YKL40 | 8 | 467 | 220 | 0.88 [0.57; 1.19] | 3 | 221 | 80 | 0.83 [0.56; 1.10] | 4 | 253 | 98 | 0.71 [0.43; 1.00] |
| CHI3L2 | - | - | - | - | - | - | - | - | 2 | 112 | 29 | 0.85 [0.26; 1.44] |
| GFAP | 5 | 204 | 149 | 0.41 [0.01; 0.81] | - | - | - | - | - | - | - | - |
| t-tau | 5 | 306 | 303 | 0.14 [-0.35; 0.64] | - | - | - | - | 2 | 233 | 80 | 0.54 [0.28; 0.80] |
| p-tau181 | 5 | 418 | 382 | -0.27 [-0.61; 0.06] | 2 | 277 | 102 | 0.11 [-0.12; 0.34] | 3 | 313 | 126 | 0.14 [-0.07; 0.35] |
| p-tau/t-tau | 2 | 203 | 133 | -0.51 [-1.59; 0.57] | 2 | 277 | 102 | -1.06 [-1.34; -0.78] | 2 | 277 | 102 | -1.06 [-1.34; -0.78] |
| Aβ42 | 3 | 211 | 240 | 0.34 [-0.24; 0.92] | - | - | - | - | - | - | - | - |
| sTREM2 | 5 | 189 | 124 | 0.53 [0.21; 0.85] | - | - | - | - | - | - | - | - |
| Ferritin | - | - | - | - | - | - | - | - | 3 | 334 | 184 | 0.35 [0.17; 0.53] |

###### Supplementary Table S2: Covariates included in multivariable Cox regression models

| **Study** | **Covariates included in multivariable Cox models** | |
| --- | --- | --- |
| **Blood NfL** |  | |
| Benatar 2024 | age at onset, ΔFRS, bulbar onset, diagnostic delay, SVC percent predicted, El Escorial definite ALS, presence of FTD, and presence of a C9orf72 repeat expansion | |
| Falzone 2022 | age at venipuncture, Diagnostic delay, progression rate, phenotype (spinal vs bulbar), C9orf72 expansion | |
| Gille 2019–2 | age at sampling, gender, FVC, site of onset, disease progression rate, UMN/LMN degeneration extent, C9orf72 status, FTD status | |
| Kläppe 2024 | age, sex, BMI, site of onset, El Escorial classification, diagnostic delay, baseline ALSFRS-R, progression rate, signs of FTD | |
| Puentes 2021 | age at baseline, gender, ΔFRS, bulbar onset, ALSFRS-R at Visit1, C9orf 72 mutation | |
| Shi 2022 | ALSFRS-r score, DPR, UMN score and cMAP amplitudes | |
| Vacchiano 2021 | age at baseline, sex, baseline ALSFRS-R score, genetic status, DPR, MRC, King’s scores. | |
| Verde 2023–1 | age at onset, site of onset, presence of C9orf72 hexanucleotide repeat expansion (HRE), disease progression rate (DPR), and presence of ALS-specific cognitive impairment according to ECAS. | |
| **Blood pNfH** |  | |
| Benatar 2024 | age at onset, ΔFRS, bulbar onset, diagnostic delay, SVC percent predicted, El Escorial definite ALS, presence of FTD, and presence of a C9orf72 repeat expansion | |
| De Schaepdryver 2019 | age at onset, presence of bulbar symptoms | |
| Falzone 2020 | age at venipuncture, diagnostic delay, progression rate, and MND phenotype. | |
| Shi 2022 | ALSFRS-r score, DPR, UMN score and cMAP amplitudes | |
| **Blood GFAP** |  | |
| Falzone 2022 | age at venipuncture, Diagnostic delay, progression rate, phenotype (spinal vs bulbar), C9orf72 expansion | |
| Mastrangelo 2023 | age at sampling, onset type, ALSFRS-R scale, FTD status, DPR | |
| **Blood CRP** |  | |
| Benatar 2024 | age at onset, ΔFRS, bulbar onset, diagnostic delay, SVC percent predicted, El Escorial definite ALS, presence of FTD, and presence of a C9orf72 repeat expansion | |
| Sun 2020 | age, sex | |
| **Blood Creatinine** |  | |
| Benatar 2024 | age at onset, ΔFRS, bulbar onset, diagnostic delay, SVC percent predicted, El Escorial definite ALS, presence of FTD, and presence of a C9orf72 repeat expansion | |
| Guo 2021 | age of onset, site of onset, diagnostic delay, BMI, ALSFRS-R score, progression rate (ΔALSFRS-R), use of riluzole, use of NIPPV, and acceptance of PEG. | |
| Morgadinho 2021 | age, gender, disease duration, onset phenotype, BMI, clinical signs of FTD | |
| Zhu 2024–2 | age, sex, BMI, site of onset, diagnostic delay and ALSFRS-R score | |
| **Blood NLR** |  | |
| Leone 2022 | age at recruitment, gender, country (Italy vs. Moldova/Romania), FVC, BMI, site of onset, and use of riluzole. | |
| Wei 2022 | age, sex, stage, phenotype, onset region, BMI, ALSFRS-R, disease duration, albumin, and HbA1c. | |
| Zhu 2024–2 | age, sex, BMI | |
| **Blood UA** |  | |
| Benatar 2024 | age at onset, ΔFRS, bulbar onset, diagnostic delay, SVC percent predicted, El Escorial definite ALS, presence of FTD, and presence of a C9orf72 repeat expansion | |
| Xu 2021 | age of onset, site of onset, BMI at baseline, ALSFRS-R score at baseline, progression rate (∆ALSFRS-R), diagnostic delay, use of riluzole, use of NIPPV, acceptance of PEG, and serum uric acid levels. | |
| **Blood CysC** |  | |
| Nagel 2023 | age, sex, diagnostic delay, site of onset, ALSFRS, BMI, self-reported DM, self-reported smoking(ever) | |
| Zhu 2024–2 | age, sex, BMI | |
| **CSF NfL** |  |  |
| Abu-Rumeileh 2020 | age, sex, FTD status (0-1) |  |
| Huang 2020 | age of disease onset, site of onset, gender |  |
| Kläppe 2024 | age, sex, BMI, site of onset, El Escorial classification, diagnostic delay, baseline ALSFRS-R, progression rate, signs of FTD |  |
| Rosén 2024 | age, sex |  |
| Shi 2022 | ALSFRS-r score, DPR, UMN score and cMAP amplitudes |  |
| Steinacker 2021 | age, sex, site of onset, progression rate |  |
| Vacchiano 2021 | age at baseline, sex, baseline ALSFRS-R score, genetic status, DPR, MRC, King’s scores. |  |
| **CSF pNfH** |  |  |
| Kläppe 2024 | age, sex, BMI, site of onset, El Escorial classification, diagnostic delay, baseline ALSFRS-R, progression rate, signs of FTD |  |
| Shi 2022 | ALSFRS-R score, DPR, UMN score and cMAP amplitudes |  |
| Steinacker 2021 | age, sex, site of onset, progression rate |  |
| **CSF CHIT1** |  |  |
| Gille 2019–1 | age at LP, disease progression rate (points/month), number of regions with UMN and LMN degeneration, FVC, definite ALS based on the revised El Escorial criteria, diagnostic delay, gender, C9orf72 status, FTD status, and bulbar onset |  |
| Kläppe 2024 | age, sex, BMI, site of onset, El Escorial classification, diagnostic delay, baseline ALSFRS-R, progression rate, signs of FTD |  |
| Steinacker 2021 | age, sex, site of onset, progression rate |  |
| **CSF YKL40** |  |  |
| Gille 2019–1 | age at LP, disease progression rate (points/month), number of regions with UMN and LMN degeneration, FVC, definite ALS based on the revised El Escorial criteria, diagnostic delay, gender, C9orf72 status, FTD status, and bulbar onset |  |
| Rosén 2024 | age, sex |  |
| **CSF MCP1** |  |  |
| Gille 2019–1 | age at LP, disease progression rate (points/month), number of regions with UMN and LMN degeneration, FVC, definite ALS based on the revised El Escorial criteria, diagnostic delay, gender, C9orf72 status, FTD status, and bulbar onset |  |
| Huang 2020 | age of disease onset, site of onset, gender |  |
| Kläppe 2024 | age, sex, BMI, site of onset, El Escorial classification, diagnostic delay, baseline ALSFRS-R, progression rate, signs of FTD |  |
